# Post-transcriptional labeling by using Suzuki–Miyaura cross-coupling generates functional RNA probes

**DOI:** 10.1093/nar/gky185

**Published:** 2018-03-13

**Authors:** Manisha B Walunj, Arun A Tanpure, Seergazhi G Srivatsan

**Affiliations:** 1 Department of Chemistry, Indian Institute of Science Education and Research (IISER), Pune Dr. Homi Bhabha Road, Pune 411008, India; 2 Department of Chemistry, University of Cambridge, Lensfield Road, Cambridge CB2 1EW, UK

## Abstract

Pd-catalyzed C-C bond formation, an important vertebra in the spine of synthetic chemistry, is emerging as a valuable chemoselective transformation for post-synthetic functionalization of biomacromolecules. While methods are available for labeling protein and DNA, development of an analogous procedure to label RNA by cross-coupling reactions remains a major challenge. Herein, we describe a new Pd-mediated RNA oligonucleotide (ON) labeling method that involves post-transcriptional functionalization of iodouridine-labeled RNA transcripts by using Suzuki–Miyaura cross-coupling reaction. 5-Iodouridine triphosphate (IUTP) is efficiently incorporated into RNA ONs at one or more sites by T7 RNA polymerase. Further, using a catalytic system made of Pd(OAc)_2_ and 2-aminopyrimidine-4,6-diol (ADHP) or dimethylamino-substituted ADHP (DMADHP), we established a modular method to functionalize iodouridine-labeled RNA ONs in the presence of various boronic acid and ester substrates under very mild conditions (37°C and pH 8.5). This method is highly chemoselective, and offers direct access to RNA ONs labeled with commonly used fluorescent and affinity tags and new fluorogenic environment-sensitive nucleoside probes in a ligand-controlled stereoselective fashion. Taken together, this simple approach of generating functional RNA ON probes by Suzuki–Miyaura coupling will be a very important addition to the resources and tools available for analyzing RNA motifs.

## INTRODUCTION

Understanding of RNA structure and function, and its use in therapeutics are greatly aided by recent developments in the nucleic acid functionalization strategy based on bioorthogonal chemical reactions (1–4). Traditional approaches like solid-phase ON synthesis and enzymatic methods are very useful in installing variety of probes onto RNA for various biophysical investigations. However, in several instances, elaborate chemical manipulations to synthesize the functionalized monomers (e.g. phosphoramidites and triphosphates) and challenges associated with their incorporation (e.g. stability under reaction conditions, poor coupling and enzymatic incorporation efficiency) limit the applications of these methods (4). In this context, post-synthetic modification of RNA by using bioorthogonal reactions is proving as a valuable tool to generate functional RNA probes. In this method, an RNA ON is labeled with a small reactive handle by using solid-phase ON synthesis protocol or by using the substrate promiscuity of RNA polymerases and certain RNA processing enzymes (e.g. transferases, 4–6). Following this step, a chemoselective reaction with the cognate reactive partner is performed to introduce the desired functional modification into the RNA. Reactions like azide-alkyne cycloaddition (7–22), Staudinger ligation (21), inverse electron demand Diels–Alder (23–28), to name a few, have emerged as valuable tools to label, image and profile RNA in cell-free and cellular environments. These methods often use bulky activated building blocks (e.g. cyclooctyne, tetrazine, norbornyl etc.) to promote efficient post-synthetic reaction under mild conditions. However, the synthesis of many of these building blocks is tedious involving multiple steps, and if commercially available are very expensive (29–31). Therefore, establishment of new post-synthetic RNA modification strategies that allow direct introduction of various functionalities by using easily accessible tags and reporters remains a high priority.

In this regard, Pd-mediated C-C bond formation, which is applied in almost all facets of chemistry, is proving useful as a valuable chemoselective transformation for synthetic modification of biomacromolecules (32–36). This has been possible due to the development of new Pd-ligand catalytic systems, which appreciably accelerate the coupling reaction in aqueous buffer (37–39). Manderville group first demonstrated the usefulness of Suzuki–Miyaura reaction in the post-synthetic functionalization of DNA oligonucleotides (ON, (40)). ONs containing 8-bromoguanosine were reacted with arylboronic acids in the presence of a catalytic system made of Pd(OAc)_2_ and a water-soluble triphenylphosphan-3,3′,3″-trisulfonate ligand, which was used previously for nucleotide modification (41,42). Using a similar method, a diarylethene photoswitch capable of undergoing reversible electrocyclic rearrangement was introduced into DNA ONs (43). This catalytic system requires elevated temperature (>70°C), long reaction time and alkaline conditions to generate coupled ON products in moderate yields. Meantime, Davis group used a combination of Pd(OAc)_2_ and 2-aminopyrimidine-4,6-diol (ADHP) or dimethylamino-substituted ADHP (DMADHP), which was originally developed for labeling proteins by Suzuki and Sonogashira reactions (44–46), to step up a milder route to directly install functional labels onto DNA ONs by using Suzuki–Miyaura reaction (47).

**Figure 1.**
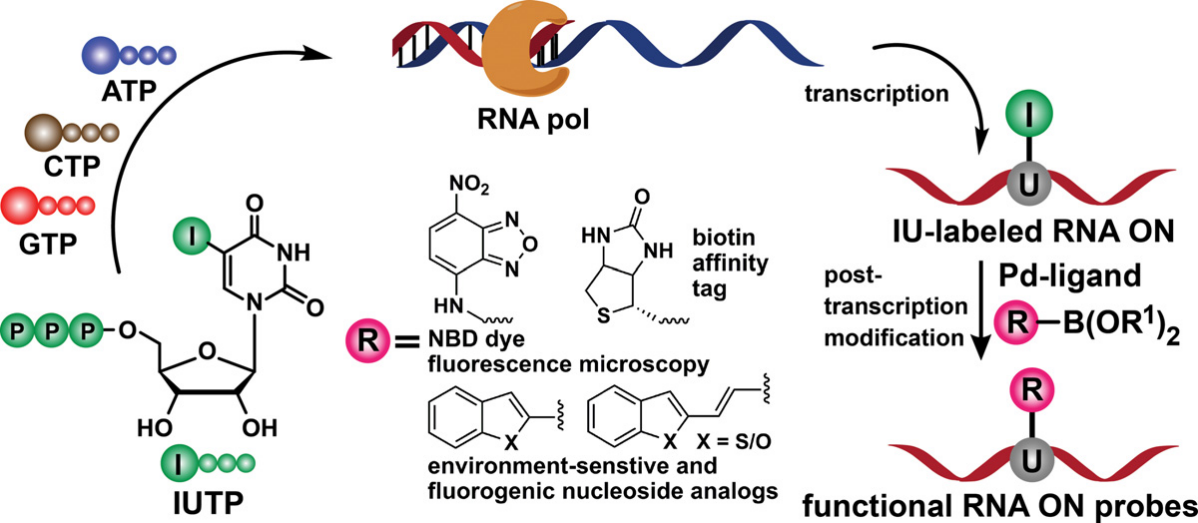
Design showing the post-transcriptional functionalization of iodouridine-labeled RNA transcripts by using Suzuki–Miyaura cross-coupling reaction to generate RNA labeled with functional probes.

Despite these successes with protein and DNA, functionalization of RNA by Pd-mediated coupling reactions remains a major challenge as methods developed for protein and DNA mostly do not work for RNA due to inherently low stability of RNA (28,48–49). Therefore, we embarked on establishing a milder and efficient method to modify RNA by first incorporating a halogenated nucleotide analog into RNA by transcription reaction, followed by a post-transcriptional Suzuki–Miyaura reaction in the presence of a cognate reactive partner labeled with a desired biophysical reporter or tag (Figure 1). Here, we demonstrate a post-transcriptional modification method to generate functional RNA probes by using Suzuki–Miyaura reaction under benign conditions (37°C and pH 8.5). This method is modular, and offers direct access to RNA labeled with fluorogenic environment-sensitive nucleoside analogs for nucleic acid structure and recognition analysis, fluorescent probes for microscopy and an affinity tag for pull-down and immunoassay (Figure 1).

## MATERIALS AND METHODS

Experimental procedure for the synthesis of 5-Iodouridine triphosphate (IUTP) and boronic ester substrates are provided in the Supplementary Data.

### Incorporation of IUTP by *in vitro* transcription reaction

#### Radiolabel experiment

The promoter-template duplexes (5 μM) were assembled by heating a 1:1 mixture of DNA promoter of T7 RNA polymerase consensus sequence and DNA ON templates **T1**–**T5** in annealing buffer (10 mM Tris–HCl, 1 mM ethylenediaminetetraacetic acid (EDTA), 100 mM NaCl, pH 7.8) at 90°C for 3 min. The solution was allowed to attain room temperature slowly and then kept in an ice bath for 20 min followed by storing at −40°C. The transcription reactions were carried out at 37°C in 40 mM Tris–HCl buffer (pH 7.8) containing 250 nM annealed promoter-template duplexes, 10 mM MgCl_2_, 10 mM NaCl, 10 mM of dithiothreitol (DTT), 2 mM spermidine, 1 U/μl RNase inhibitor (Riboblock), 1 mM guanosine triphosphate (GTP), cytidine triphosphate (CTP), uridine triphosphate (UTP) and or IUTP **2**, 20 μM adenosine triphosphate (ATP), 5 μCi α-^32^P ATP and 3 U/μl T7 RNA polymerase in a 20 μl reaction volume. The reaction was quenched after 3.5 h by adding 20 μl of loading buffer (7 M urea in 10 mM Tris–HCl, 100 mM EDTA, 0.05% bromophenol blue, pH 8). Each sample was heated for 3 min at 75°C and then cooled in an ice bath. The samples (4 μl) were loaded on an 18% denaturing polyacrylamide gel and were electrophoresed. The radioactive bands were phosphorimaged and then quantified by using GeneTools software from Syngene to determine the relative transcription efficiencies. Percentage incorporation of IUTP **2** is reported with respect to transcription efficiency in the presence of natural NTPs. All reactions were performed in duplicate and the errors in yields were <2%.

#### Large-scale transcription reaction using template T1 and IUTP 2

Transcription reaction was performed in 250 μl reaction volume using 2 mM of ATP, GTP, CTP and IUTP **2**, 20 mM MgCl_2_, 10 mM DTT, 0.40 U/μl RNase inhibitor (Riboblock), 300 nM promoter-template duplex and 800 units of T7 RNA polymerase. The reaction mixture was incubated at 37°C for 6 h. The reaction volume was made one-third by speed vac followed by addition of 50 μl denaturing loading buffer (7 M urea in 10 mM Tris–HCl, 100 mM EDTA, pH 8) and the sample was loaded on a preparative 20% denaturing polyacrylamide gel. After running the gel for 6 h, appropriate band was marked by UV shadowing and excised from the gel. In order to isolate the RNA, the band was extracted with 0.3 M sodium acetate and desalted using a Sep-Pak classic C18 cartridge. Under these conditions, an average of 10 nmole of the iodo-modified RNA transcript **4** was isolated (*ϵ_260_* = 84300 M^−1^cm^−1^). The purity and identity of the iodo-labeled RNA transcript was confirmed by matrix-assisted laser desorption/ionization-time of flight (MALDI-TOF) mass analysis (Supplementary Figure S2).

### Suzuki–Miyaura reaction between transcript 4 and boronic ester (9−11, 15−17)/boronic acid (12−14)

#### Preparation of Pd(OAc)_2_(L)_2_ complex

To L1 (65 mg, 0.5 mmol) or L2 (78 mg, 0.5 mmol) in a 5 ml round bottom flask was added autoclaved H_2_O (3 ml) and NaOH (10 M stock, 100 μl, 1 mmol). The solution was stirred for 5 min at RT to obtain a clear solution. Then Pd(OAc)_2_ (55 mg, 0.25 mmol) was added and the mixture was stirred at 65°C for 1 h. Solution was taken in a 5 ml volumetric flask and the volume was adjusted to 5 ml with autoclaved H_2_O to give a final stock solution of 50 mM.

#### Analytical-scale reaction with boronic ester 9 and 10

To a solution of iodo-modified RNA transcript **4** (200 μM, 1 equivalent) in 10 μl of Tris–HCl buffer (50 mM, pH 8.5) was added boronic ester **9** or **10** (50 or 100 equivalent) dissolved in dimethyl sulfoxide (DMSO). The coupling reaction was initiated by adding Pd(OAc)_2_(L1)_2_ catalyst (1 or 2 equivalent). Final reaction volume was 50 μl containing 20% DMSO (v/v). The reaction mixture was incubated at 37°C in thermoshaker. Aliquots of reaction mixture (16 μl) were taken at 3, 6 and 9 h, and 10 μl of denaturing loading buffer (7 M urea in 10 mM Tris–HCl, 100 mM EDTA, pH 8) was added to each aliquot. Samples were loaded on an analytical 20% denaturing polyacrylamide gel. Bands corresponding to the products were visualized by UV-shadowing method (short wave UV 254 nm and long wave UV 365 nm).

#### Large-scale reaction

To a solution of iodo-modified RNA transcript **4** (5 nmol, 100 μM, 1 equivalent) in 10 μl of Tris–HCl buffer (50 mM, pH 8.5) was added boronic acid/ester dissolved in DMSO (250 nmol, 5 mM, 50 equivalent). The reaction was initiated by adding catalyst Pd(OAc)_2_(L)_2_ (10 nmol, 0.2 mM, 2 equivalent). The final reaction volume was adjusted to 50 μl with water and DMSO such that the percentage of DMSO was 20% v/v. The reaction was incubated at 37°C for 6−12 h. The reaction mixture was filtered using a spin filter (0.45 μm pore size) and was further washed with 40 μl of water. The filtrate was analyzed by RP-HPLC (Phenomenex-Luna C18 column, 250 × 4.6 mm, 5 micron). Mobile phase A: 50 mM triethylammonium acetate (TEAA) buffer (pH 7.0), mobile phase B: acetonitrile. Flow rate: 1 ml/min. Gradient: 0−30% B in 35 min, 30−100% B in 10 min and 100% B for 5 min. The run was monitored by UV absorption at 260 nm. Each peak was collected and freeze-dried. Similar procedure was used for post-transcriptional Suzuki coupling between IU-labeled transcripts **19** and **20** with ester **17**.

#### Enzymatic digestion of Suzuki-coupled RNA ON product 17a′

A total of 3 nmol of RNA ON **17a′** was treated with snake venom phosphodiesterase I (10 μl, 0.01 U), calf intestinal alkaline phosphatase (10 μl, 1 U/μl) and RNase A (5 μl, 0.25 μg), MgCl_2_ (20 μl, 40 mM), 10 μl of dephosphorylation buffer (50 mM Tris–HCl buffer, 40 mM MgCl_2_, 0.1 mM EDTA pH 8.5) in a total volume of 100 μl for 12 h at 37°C. Subsequently, RNase T1 (2 μl, 0.2 U/μl) was added and the sample was incubated for another 4 h at 37°C. The ribonucleoside mixture obtained from the digest was analyzed by RP-HPLC using Phenomenex-Luna C18 column (250 × 4.6 mm, 5 micron) at 260 and 338 nm. Mobile phase A: 50 mM TEAA buffer (pH 7.0), mobile phase B: acetonitrile. Flow rate: 1 ml/min. Gradient: 0−10% B in 20 min, 10−100% B in 10 min, 0−100% B in 5 min. The fraction corresponding to the individual ribonucleosides was collected and analyzed by mass spectrometry, which also confirmed the identity of the natural ribonucleosides (C, G and A) and Suzuki-coupled ribonucleoside **18** (*trans* form).

### Fluorescence of 5-(benzofuran-2-yl)vinyl uridine-modified RNA ONs obtained by post-transcriptional Suzuki coupling

RNA ONs **17a′, 19a′** and **20a′** (10 μM) were hybridized to custom DNA ON (11 μM) by heating a mixture (1:1.1) of the ON in 20 mM cacodylate buffer (pH 7.1, 500 mM NaCl, 0.5 mM EDTA) at 90°C for 3 min. Samples were cooled slowly to room temperature and incubated in crushed ice for 2 h. Samples were diluted to give a final concentration of 0.5 μM with respect to **17a′, 19a′** and **20a′** in cacodylate buffer. Samples were excited at 350 nm with an excitation slit width of 4 nm and emission slit width of 5 nm. Fluorescence experiments were performed in triplicate in a micro fluorescence cuvette (Hellma, path length 1.0 cm) on a Horiba JobinYvon, Fluorolog-3 at 20°C.

## RESULTS AND DISCUSSION

### Synthesis of 5-Iodouridine-labeled RNA ON transcripts

Base-functionalized nucleoside analogs containing fluorescent, isotope, heavy atom or spin labels serve as excellent tools for biophysical investigation of nucleic acid structure, dynamics and function (50–61). Many such modified nucleoside and nucleotide substrates, suitable for incorporation into ONs, are synthesized from halogenated nucleosides and nucleotides (e.g. iodo-labeled substrates) by using Pd-catalyzed cross-coupling reaction as the key step (62–64). However, necessity to prepare individual substrates, and the challenges associated with their synthesis and incorporation (*vide supra*) can be circumvented by developing a modular post-synthetic RNA labeling method, which would allow direct installation of the probes by Suzuki–Miyaura reaction between iodo-labeled RNA ONs with various easily accessible boronic acid/ester substrates (Figure 1).

**Figure 2.**
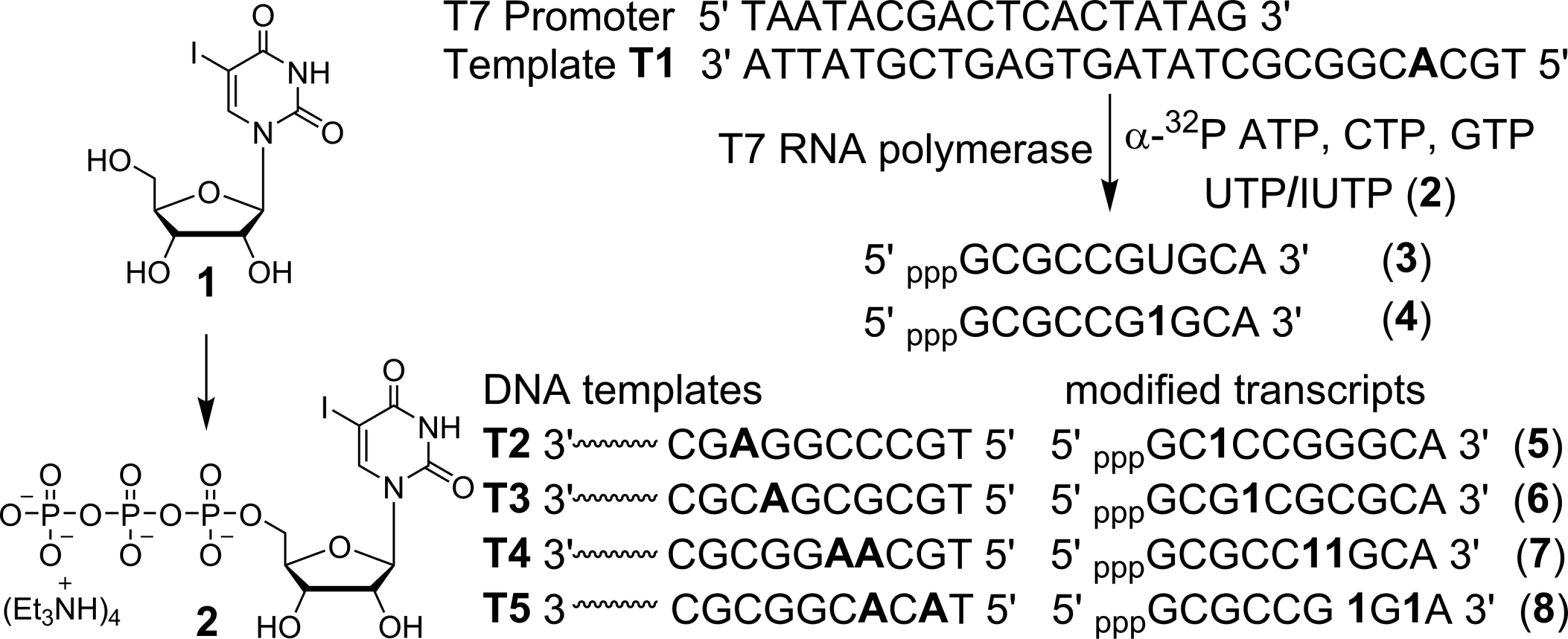
Incorporation of IUTP **2** (prepared from IU **1**) into RNA ONs by *in vitro* transcription reactions using T7 RNA polymerase and templates **T1**−**T5**. Transcripts **4**−**8** containing IU label at different sites are shown.

In order to setup an RNA functionalization method by Suzuki–Miyaura reaction, we chose to incorporate 5-iodouridine 5′-triphosphate (IUTP **2**) into RNA transcripts by *in vitro* transcription reaction (65,66). Iodo-modified nucleoside phosphoramidites can also be used to incorporate the halogen label into RNA ONs by solid-phase method. IUTP was prepared by phosphorylating IU (**1**) using POCl_3_ and *bis*-tributylammonium pyrophosphate (Figure 2). The efficiency of IUTP incorporation by bacteriophage T7 polymerase was evaluated by performing *in vitro* transcription reactions with a series of T7 promoter-template DNA duplexes (Figure 2). The templates were designed to guide the incorporation of monophosphate of IUTP into RNA at one or two sites. The templates also contained a single dT residue at the 5′-end of the coding region so that a reaction performed in the presence of UTP/IUTP, GTP, CTP and α-^32^P ATP, if successful, would result in the formation of the full-length transcripts containing a radioactive α-^32^P A label at the 3′-end.

**Figure 3.**
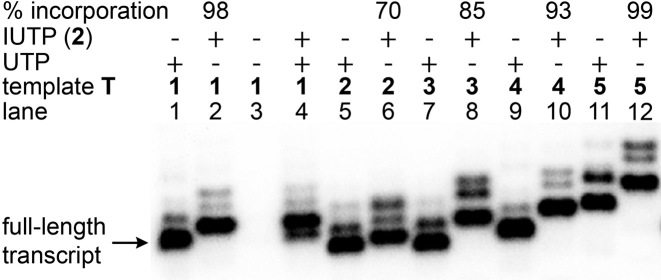
Phosphor image of transcripts obtained by *in vitro* transcription of DNA templates **T1**−**T5** in the presence of UTP/IUTP **2**. Incorporation efficiency of **2** is reported with respect to a control reaction with UTP. Trace amounts of non-templated products are formed along with full-length transcripts. For complete gel picture see Supplementary Figure S1.

Reactions performed with template **T1** and UTP/IUTP produced full-length transcripts **3** and **4**, respectively, with excellent efficiency and comparable yields (98%, Figure 3, lanes 1 and 2). Slower mobility of **4** compared to **3** indicated the incorporation of modified U into transcript **4**. The labeling of IU in the full-length transcript was confirmed by mass measurement of the purified transcript prepared from a large-scale reaction (Supplementary Figure S2). A control reaction in the absence of UTP and IUTP did not yield full-length transcript, indicating that there was no misincorporation during the transcription process (lane 3). Interestingly, in a reaction containing 1:1 molar ratio of UTP and IUTP, the RNA polymerase preferentially incorporated IUTP over UTP (lane 4). Reactions with other templates (**T2**−**T5**) indicated that IU can be introduced near the promoter region and at more than one site with very good efficiency (lanes **5**−**12**).

### Post-transcriptional Suzuki–Miyaura cross-coupling

#### Optimization of coupling reaction conditions

**Figure 4.**
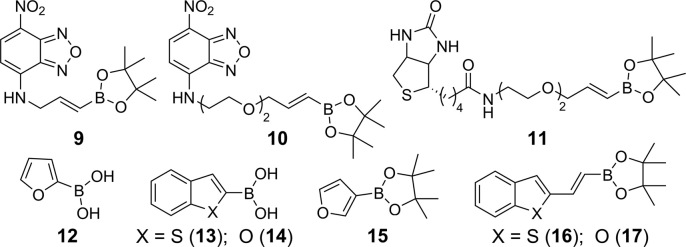
Substrates used in post-transcriptional Suzuki–Miyaura coupling.

**Figure 5.**
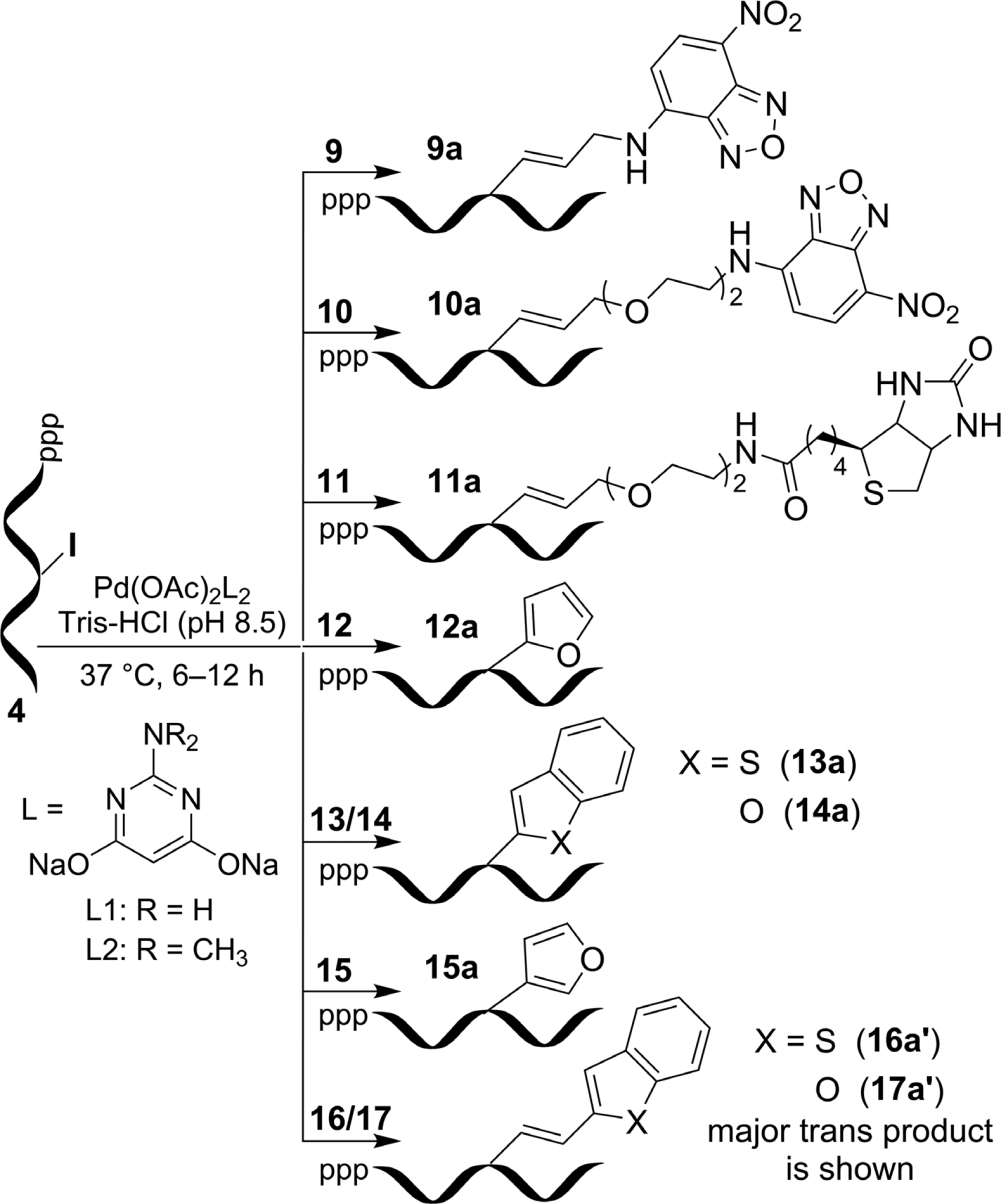
Post-transcriptional chemical functionalization of IU-labeled RNA transcript **4** with substrates **9**−**17** by using Suzuki–Miyaura reaction.

**Figure 6.**
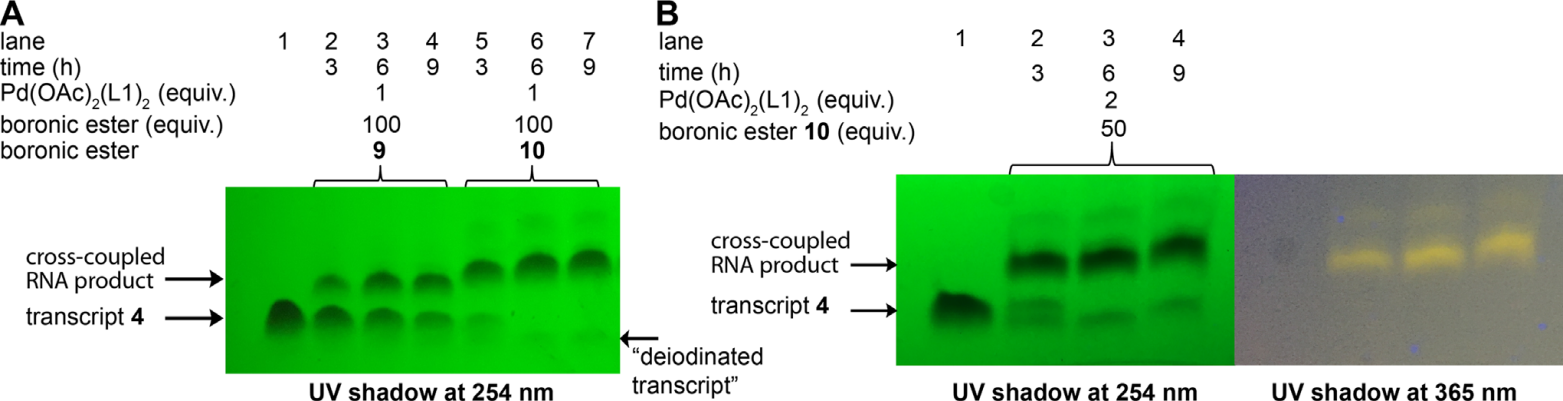
(**A**) Suzuki reaction on iodo-labeled RNA ON **4** using 1 equivalent of Pd-catalyst and 100 equivalent of boronic ester **9/10**. UV-shadow of the gel (short wave UV, 254 nm). (**B**) Suzuki reaction on IU-labeled RNA ON **4** using 2 equivalent of Pd-catalyst and 50 equivalent of boronic ester **10**. UV-shadow of the gel at 254 nm (left) and at 365 nm (right).

IU-labeled RNA ON **4** was subjected to Suzuki–Miyaura cross-coupling at different stoichiometries of the substrate and reagents so as to achieve good conversion with minimum degradation of the coupled product. We preferred to use a combination of Pd(OAc)_2_ and ADHP (L1) or DMADHP (L2) as this system has been shown to be efficient in the Pd-mediated functionalization of protein and DNA (44–47). Coupling reaction was performed by incubating **4** (1 equivalent) with Pd(OAc)_2_L1_2_ (1 equivalent) and nitrobenzofurazan (NBD)-labeled boronic ester **9/10** (commonly used dye in fluorescence imaging, 100 equivalent) in Tris–HCl buffer (50 mM, pH 8.5) at 37°C (Figures 4 and 5). Aliquots of reaction mixture after 3, 6 and 9 h were resolved by analytical polyacrylamide gel electrophoresis under denaturing conditions, and analyzed by UV-shadowing. Rewardingly, reactions with boronic esters **9** and **10** resulted in the formation of respective coupled RNA product, which migrated slower compared to the substrate **4** (Figure 6A). A reaction with NBD dye (**10**) attached to boronic ester via a longer linker showed almost complete consumption of the substrate in 6 h as compared to **9**, which remained partially consumed after 9 h. When the amount of boronic ester **10** was reduced to 50 equivalents, the reaction required a slightly higher loading of Pd-L1 (2 equivalent) to effect the coupling in 6 h (Figure 6B). UV-shadowing the gel at longer wavelength (∼365 nm) further confirmed the fluorescence labeling of RNA with NBD. Importantly, under these conditions we observed no detectable degradation of the RNA product.

#### Post-transcriptional coupling of RNA with fluorescent and affinity tags

Based on these preliminary results, transcript **4** (5 nmol, 1 equivalent) was then subjected to post-transcriptional coupling reaction with boronic esters **9**−**11** (50 equivalent) using Pd-L1/L2 (2 equivalent) catalytic systems. The reaction was monitored by High performance liquid chromatography (HPLC), and the peak corresponding to the product was isolated and characterized by mass analysis (Supplementary Figure S3 and Table S1). While UV-shadowing of the gel gave a qualitative understanding of the coupling reaction, HPLC analysis gave a better understanding of the reaction in terms of efficiency and isolated yields (Table 1). A reaction with NBD-boronic ester **9**, containing a short linker, in the presence of L1 gave 28% of the fluorescent RNA product **9a** in 12 h. However, a reaction with NBD-boronic ester **10**, containing a longer linker, afforded 30% of the fluorescent RNA product **10a** within 6 h. Under similar conditions, biotin-coupled RNA product **11a** was isolated in 28% yield from a reaction with biotinylated boronic ester substrate **11**. HPLC chromatogram of reactions with substrates **9**−**11** revealed the formation of noticeable amount of deiodinated transcript along with unreacted transcript **4**, and hence, longer reaction times were not attempted with these substrates (Supplementary Figure S3). Reactions using Pd-L2 system for above substrates were found to be less efficient as compared to Pd-L1 combination (Table 1).

**Table 1.** Yields of Suzuki–Miyaura cross-coupled RNA ON products obtained by post-transcriptional chemical modification of IU-labeled RNA ON transcripts^a^

Entry	RNA ON	Boronate ester substrate	Reaction time (h)	Cross-coupled product	*ϵ_260_*M^−1^cm^−1b^	Isolated yield (nmol) (with ligand L1)	Isolated yield (%) (with ligand L1)	Isolated yield (%) (with ligand L2)
1	**4**	**9**	12	**9a**	84 740	1.4	28	11
2	**4**	**10**	6	**10a**	84 740	1.5	30	24
3	**4**	**11**	6	**11a**	84 740	1.4	28	23
4	**4**	**12**	6	**12a**	92 420	1.8	36	
5	**4**	**13**	6	**13a**	90 340	1.5	30	
6	**4**	**14**	6	**14a**	98 553	1.6	32	
7	**4**	**15**	6	**15a**	92 420	2.5	50	25
8	**4**	**16**	6	**16′ (16a″)** ^c^	85 020	3.1 (0.9)	62 + 18 = 80	46
9	**4**	**17**	6	**17a′ (17a″)** ^c^	85 400	2.6 (0.5)	52 + 10 = 62	43
10	**19**	**17**	12	**19a′ (19a″)** ^c^	79 400	0.9 (0.6)	18 + 12 = 30	
11	**20**	**17**	6	**20a′ (20a″)** ^c^	91 800	1.8 (0.8)	36 + 16 = 52	

^a^All reactions were performed on a 5 nmole scale of IU-labeled RNA transcripts. Yields reported are with respect to the RNA products isolated after HPLC purification. Concentration and yield of the product was calculated using the molar absorption coefficient (*ϵ_260_*) of the RNA product. See Supplementary Figures S3, 4 and 6, and Table S1 for mass spectra and data.

^b^ *ϵ_260_* of coupled RNA ON products was determined by using OligoAnalyzer 3.1. In case of **9a**−**11a**, *ϵ_260_*of 5-vinyluridine (28) was used in place of uridine. For **12a**−**15a**, *ϵ_260_* of corresponding 5-heterocycle-coupled uridine was used in place of uridine (76–78). For coupled RNA ON products using boronic esters **16** and **17**, *ϵ_260_*of 5-(benzothiophen-2-yl)vinyl uridine (3820 M^−1^cm^−1^) and 5-(benzofuran-2-yl)vinyl uridine (4200 M^−1^cm^−1^) was determined, and used in place of uridine.

^c^ **16a′, 17a′, 19a′** and **20a′** represent the *trans* isomer of cross-coupled product (major). **16a″, 17a″, 19a″** and **20a″** given in parenthesis represent the *‘*cis’ isomer of cross-coupled product (minor). Isolated yields in nmoles and percentage for *trans* and ‘cis*’* isomers products are also given.

#### Post-transcriptional coupling generates RNA labeled with fluorogenic environment-sensitive nucleoside analogs

Several examples including the ones reported from our laboratory indicate that responsive fluorescent nucleoside analogs can be assembled by conjugating heterocyclic rings onto nucleobases (67–75). Such nucleosides incorporated into ONs by chemical or enzymatic means serve as excellent probes for studying nucleic acid structure and function, and in diagnostic applications. These strategies usually use modified nucleoside phosphoramidites or triphosphates, which involve elaborate chemical manipulations. Moreover, in several instances, the substrates (i) show poor coupling efficiency, (ii) do not survive the conditions used in the solid-phase protocols and (iii) are not efficiently incorporated by polymerases (4). In this context, post-transcriptional coupling of IU-labeled RNA ONs with heterocycle-containing boronic acids/esters should provide direct access to RNA functionalized with responsive nucleoside probes. This approach will avoid cumbersome synthesis and challenges in the incorporation of individual modified amidites and nucleotides.

First we focused on functionalizing RNA with some known environment-sensitive base-modified fluorescent nucleoside analogs. IU-labeled RNA ON **4** was reacted with commercially available heteroaryl boronic acid derivatives **12**−**15** in the presence of Pd-L1 (Figures 4 and 5). Reactions with these substrates gave fluorescent RNA ONs (**12a**−**15a**) labeled with furan-, benzothiophene- and benzofuran-modified uridine (Table 1; Supplementary Figure S4 and Table S1). These analogs have been successfully used in assays to probe nucleic acid structure, lesion, recognition and in setting up screening platforms (76–80).

Next, we sought to use this labeling approach to introduce new fluorescent nucleoside modifications into RNA ONs. Although predicting the fluorescence outcome based on the structure is difficult, we envisioned that coupling heterocycles onto nucleosides via an extended π system may impart interesting photophysical features to otherwise nonemissive nucleosides (81,82). In this regard, we chose to couple easily synthesizable heteroarylvinyl boronic esters **16** and **17** with IU-labeled RNA ON **4** (Figure 4). Rewardingly, these substrates underwent facile coupling reaction to produce RNA ONs containing 5-(benzothiophen-2-yl)vinyl uridine and 5-(benzofuran-2-yl)vinyl uridine, respectively, in good yields (Figure 5, Table 1 and Supplementary Table S1). The HPLC chromatogram of the reaction revealed the presence of major (designated as **16a′** and **17a′**) and minor (designated as **16a″** and **17a″**) peaks having the same mass; probably corresponding to *trans* and *cis* isomers, respectively (Supplementary Figure S5, Figure S6 and Table S1). In order to ascertain the isomeric identity of the products formed, the major peak (**17a′**) was isolated and digested using a cocktail of enzymes, which would generate individual ribonucleosides. The HPLC chromatogram of the digest revealed the presence of native ribonucleosides (C, G and A) and *trans* form of 5-(benzofuran-2-yl)vinyl uridine, which matched well with the retention time of the authentic *trans* isomer (**18**) obtained from a control reaction between IU and ester **17** (Supplementary Figure S7). Remarkably, reaction in the presence of Pd-L2 catalytic system yielded only the *trans* products **16a′** and **17a′**, suggesting that this post-transcriptional modification is an example of a ligand-controlled stereoselective alkenylation process (Table 1 and Supplementary Figure S5).

A 5 nmole reaction scale gave 1.4−4 nmole of the coupled RNA product, which is sufficient for subsequent biophysical analysis (Table 1). Similar yields are reported for post-synthetic click functionalization of RNA ONs (12,17,21). It is worth mentioning here that dehalogenation is a common side reaction in Pd-catalyzed cross-coupling reactions, including Suzuki–Miyaura reaction. The extent of deiodination of the substrate, and hence, the reduction in the reaction efficiency is known to vary with the reaction temperature, nature of the catalyst-ligand system and loading, nature of boronic acid/ester substrates, solvent/buffer conditions and reaction time (40,43,47). Hence, for a given boronic acid/ester substrate the deiodination can be potentially minimized by optimizing the reaction conditions in terms of catalyst loading and reaction time. In case more amount of the RNA product is required then a batch reaction is recommended, which can be pooled before purification, thereby avoiding multiple HPLC runs (data not shown). Control reactions with ON **4** in the presence of boronic acids/esters or Pd(OAc)_2_ or Pd-ligand alone did not yield the coupled product (Supplementary Figure S8). Similarly, an unmodified RNA ON **3** did not react under the coupling reaction conditions. These results indicate that Suzuki coupling on IU-labeled RNA is highly chemoselective.

#### Fluorogenic nature and environment-sensitivity of the coupled RNA products

**Figure 7.**
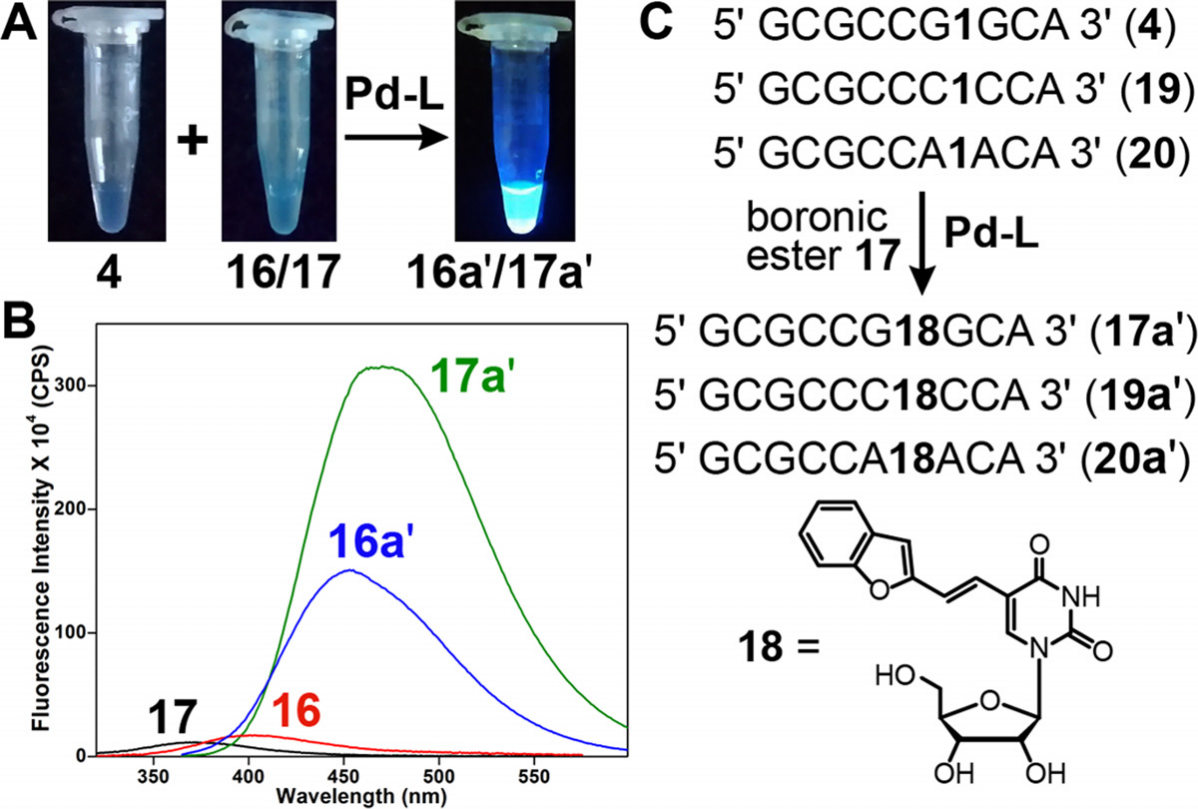
(**A**) Image showing the fluorogenic Suzuki coupling of IU-labeled transcript **4** with boronic esters **16** and **17**. The samples were irradiated using 365 nm light source. (**B**) Emission spectra (1 μM) of substrates (very weakly emissive) and RNA ON products **16a′** and **17a′** (highly emissive). (**C**) Synthesis of RNA ONs **17a′, 19a′** and **20a′**, containing emissive nucleoside **18** in-between different flanking bases, from respective iodo-labeled RNA ON transcripts by post**-**transcriptional Suzuki coupling.

Another very important and useful feature of this post-synthetic Suzuki coupling reaction is that it is fluorogenic in nature. Coupling of nonemissive IU-labeled RNA **4** with very weakly emissive boronates **16** and **17** gave highly fluorescent RNA products **16a′** and **17a′**, respectively (Figure 7A and B). In particular, 5-(benzofuran-2-yl)vinyl uridine (**18**)-modified RNA ON **17a′** displayed more than 25-fold enhancement in fluorescence intensity as compared to boronic ester **17**. The environment-sensitivity of this fluorescent label was evaluated at the nucleoside level (**18**) and by using **18**-containing RNA ONs (**17a′, 19a′** and **20a′**, Figure 7C). These ONs in which the emissive nucleoside is placed in-between different flanking bases were prepared by post-transcriptional Suzuki coupling of IU-labeled RNA ONs **4, 19** and **20**, respectively, with ester **17**.

The ground-state electronic spectrum of **18** was not significantly affected by changes in solvent polarity. However, the nucleoside exhibited excellent fluorescence solvatochromism, wherein the emission maximum, Stokes shift, intensity and quantum yield were significantly influenced by changes in the polarity of the medium (Supplementary Figure S9 and Table S3). In water, **18** displayed a weak fluorescence band (*λ*_em_ = 483 nm) corresponding to a quantum yield of 0.028. As the polarity of the medium was decreased from water to methanol to dioxane, a significant enhancement in fluorescence efficiency (5-fold) accompanied by a blue-shifted emission maximum was observed (*λ*_em_ = 427 nm in dioxane). Encouraged by these results, we next sought to study the responsiveness of the nucleoside analog to changes in neighboring base environment.

**Figure 8.**
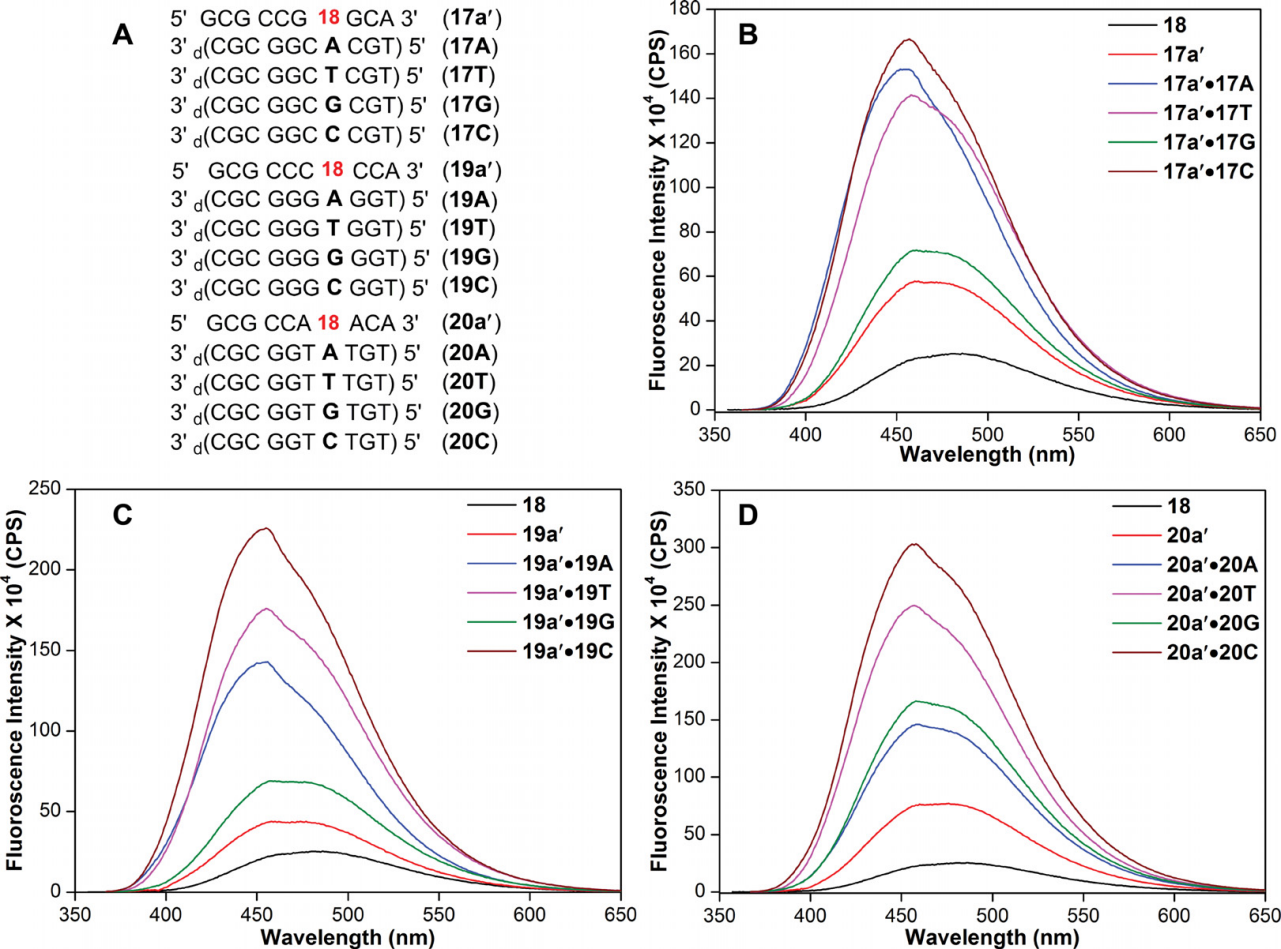
(**A**) Sequence of **18**-labeled RNA ONs **17a′, 19a′** and **20a′** and custom DNA ONs. RNA ONs were hybridized to DNA ONs such that the emissive nucleoside was placed opposite to complementary base and mismatched bases. For example, hybridization of RNA ON **17a′** with DNA ONs **17A, 17T, 17G** and **17C** will place **18** opposite to complementary base dA and mismatched bases dT, dG and dC, respectively. (**B**−**D**) Emission spectra of RNA ONs and corresponding duplexes.

RNA ONs **17a′, 19a′** and **20a′** were hybridized with DNA ONs such that the emissive analog **18** was paired opposite to complementary or mismatched bases (Figure 8A). Typical of a responsive nucleoside probe, the emission maximum and intensity of the nucleoside were found to be sensitive to neighboring base environment (Figure 8). **18** incorporated into single stranded RNA ONs and then into duplexes (matched or mismatched) showed a progressing increase in fluorescence intensity as compared to the free nucleoside analog. Notably, the emission maximum of duplexes (∼455 nm) was significantly blue-shifted as compared to the nucleoside (483 nm). The enhancement in fluorescence and blue-shifted emission indicate that the micropolarity around the emissive analog in duplexes is significantly lower than water. A comparison of emission maximum of the free nucleoside analog in different solvents and in duplexes suggest that the modification at the 5-position of uridine, which is projected in the major groove, experiences a polarity more close to methanol (Supplementary Table S3). This result is consistent with the major groove polarity of the duplexes reported in the literature (83,84). Further, among the duplexes, the emissive analog placed in the vicinity of guanine showed lower fluorescence intensity as compared to other bases, which is likely due to the known quenching effect of guanine by electron transfer process (85). Taken together, these results underscore the potential of post-synthetic Suzuki coupling reaction in directly accessing RNA ONs labeled with fluorogenic and environment-sensitive nucleosides.

Pd contamination in the labeled RNA product could potentially interfere with its application in cell-based and *in vivo* experiments due to the toxicity of Pd (86). In the Suzuki-based protein labeling method, Davis *et al*. observed loss in signal in the mass spectra due to non-specific coordination of Pd to the protein (87). This effect was significantly reduced by using 3-mercaptopropionic acid as a scavenger, which strongly binds to Pd as compared to the protein. However, when labeling DNA by Suzuki coupling such an effect was not observed (47). Similarly, in our reaction conditions and purification method, we did not observe loss of mass signal from Suzuki-labeled RNA ON products. Nevertheless, it is suggested that a Pd scavenger can be used when preparing labeled RNA ONs for *in vivo* and therapeutic applications.

## CONCLUSION

We have established an efficient method to label RNA ONs with functional probes by conceiving post-transcriptional Suzuki–Miyaura cross-coupling reaction under biocompatible conditions. This direct RNA labeling method can be used to install commonly used biophysical reporters as well as generate RNA ONs labeled with new fluorogenic and environment-sensitive nucleoside probes in a ligand-controlled stereoselective fashion. Our results demonstrate that this RNA bioconjugation approach based on Suzuki–Miyaura coupling is a very powerful tool, which will complement existing methods to functionalize and study RNA ON motifs.

## Supplementary Material

Supplementary Data

## References

[1] El-Sagheer A.H., Brown T. Click nucleic acid ligation: applications in biology and nanotechnology. Acc. Chem. Res. 2012; 45:1258–1267. 22439702 10.1021/ar200321n PMC3423825

[2] Holstein J.M., Rentmeister A. Current covalent modification methods for detecting RNA in fixed and living cells. Methods. 2016; 98:18–25. 26615954 10.1016/j.ymeth.2015.11.016

[3] Wu H., Devaraj N.K. Inverse electron-demand Diels-Alder bioorthogonal reactions. Top. Curr. Chem. 2016; 374:3. 10.1007/s41061-015-0005-z 27572986

[4] George J.T., Srivatsan S.G. Posttranscriptional chemical labeling of RNA by using bioorthogonal chemistry. Methods. 2017; 120:28–38. 28215631 10.1016/j.ymeth.2017.02.004

[5] Deen J., Vranken C., Leen V., Neely R.K., Janssen K.P.F., Hofkens J. Methyltransferase-directed labeling of biomolecules and its applications. Angew. Chem. Int. Ed. 2017; 56:5182–5200. 10.1002/anie.201608625 PMC5502580 27943567

[6] Kath-Schorr S. Cycloadditions for studying nucleic acids. Top. Curr. Chem. 2016; 374:4. 10.1007/s41061-015-0004-0 27572987

[7] Jao C.Y., Salic A. Exploring RNA transcription and turnover *in vivo* by using click chemistry. Proc. Natl. Acad. Sci. U.S.A. 2008; 105:15779–15784. 18840688 10.1073/pnas.0808480105 PMC2572917

[8] El-Sagheer A.H., Brown T. New strategy for the synthesis of chemically modified RNA constructs exemplified by hairpin and hammerhead ribozymes. Proc. Natl. Acad. Sci. U.S.A. 2010; 107:15329–15334. 20713730 10.1073/pnas.1006447107 PMC2932611

[9] Motorin Y., Burhenne J., Teimer R., Koynov K., Willnow S., Weinhold E., Helm M. Expanding the chemical scope of RNA:methyltransferases to site-specific alkynylation of RNA for click labelling. Nucleic Acids Res. 2011; 39:1943–1952. 21037259 10.1093/nar/gkq825 PMC3061074

[10] Willibald J., Harder J., Sparrer K., Conzelmann K.-K., Carell T. Click-modified anandamide siRNA enables delivery and gene silencing in neuronal and immune cells. J. Am. Chem. Soc. 2012; 134:12330–12333. 22812910 10.1021/ja303251f

[11] Paredes E., Das S.R. Optimization of acetonitrile co-solvent and copper stoichiometry for pseudo-ligandless click chemistry with nucleic acids. Bioorg. Med. Chem. Lett. 2012; 22:5313–5316. 22818972 10.1016/j.bmcl.2012.06.027

[12] Rao H., Tanpure A.A., Sawant A.A., Srivatsan S.G. Enzymatic incorporation of an azide-modified UTP analog into oligoribonucleotides for post-transcriptional chemical functionalization. Nat. Protoc. 2012; 7:1097–1112. 22576108 10.1038/nprot.2012.046

[13] Curanovic D., Cohen M., Singh I., Slagle C.E., Leslie C.S., Jaffrey S.R. Global profiling of stimulus-induced polyadenylation in cells using a poly(A) trap. Nat. Chem. Biol. 2013; 9:671–675. 23995769 10.1038/nchembio.1334 PMC3805764

[14] Santner T., Hartl M., Bister K., Micura R. Efficient access to 3′-terminal azide-modified RNA for inverse click-labeling patterns. Bioconjug. Chem. 2014; 25:188–195. 24358989 10.1021/bc400513z PMC3898571

[15] Samanta A., Krause A., Jäschke A. A modified dinucleotide for site-specific RNA-labelling by transcription priming and click chemistry. Chem. Commun. 2014; 50:1313–1316. 10.1039/c3cc46132g 24343756

[16] Li F., Dong J., Hu X., Gong W., Li J., Shen J., Tian H., Wang J. A covalent approach for site-specific RNA labeling in mammalian cells. Angew. Chem. Int. Ed. 2015; 54:4597–4602. 10.1002/anie.201410433 25694369

[17] Someya T., Ando A., Kimoto M., Hirao I. Site-specific labeling of RNA by combining genetic alphabet expansion transcription and copper-free click chemistry. Nucleic Acids Res. 2015; 43:6665–6676. 26130718 10.1093/nar/gkv638 PMC4538826

[18] Merkel M., Peewasan K., Arndt S., Ploschik D., Wagenknecht H.-A. Copper-free postsynthetic labeling of nucleic acids by means of bioorthogonal reactions. Chembiochem. 2015; 16:1541–1553. 26063100 10.1002/cbic.201500199

[19] Holstein J.M., Anhäuser L., Rentmeister A. Modifying the 5′-cap for click reactions of eukaryotic mRNA and to tune translation efficiency in living cells. Angew. Chem. Int. Ed. 2016; 55:10899–10903. 10.1002/anie.201604107 27511141

[20] Zheng Y., Beal P.A. Synthesis and evaluation of an alkyne-modified ATP analog for enzymatic incorporation into RNA. Bioorg. Med. Chem. Lett. 2016; 26:1799–1802. 26927424 10.1016/j.bmcl.2016.02.038 PMC4785081

[21] Sawant A.A., Tanpure A.A., Mukherjee P.P., Athavale S., Kelkar A., Galande S., Srivatsan S.G. A versatile toolbox for posttranscriptional chemical labeling and imaging of RNA. Nucleic Acids Res. 2016; 44:e16. 26384420 10.1093/nar/gkv903 PMC4737177

[22] Nguyen K., Fazio M., Kubota M., Nainar S., Feng C., Li X., Atwood S.X., Bredy T.W., Spitale R.C. Cell-selective bioorthogonal metabolic labeling of RNA. J. Am. Chem. Soc. 2017; 139:2148–2151. 28139910 10.1021/jacs.6b11401

[23] Schoch J., Ameta S., Jäschke A. Inverse electron-demand Diels–Alder reactions for the selective and efficient labeling of RNA. Chem. Commun. 2011; 47:12536–12537. 10.1039/c1cc15476a 22002170

[24] Ameta S., Becker J., Jäschke A. RNA–peptide conjugate synthesis by inverse- electron demand Diels–Alder reaction. Org. Biomol. Chem. 2014; 12:4701–4707. 24871687 10.1039/c4ob00076e

[25] Pyka A.M., Domnick C., Braun F., Kath-Schorr S. Diels−Alder cycloadditions on synthetic RNA in mammalian cells. Bioconjug. Chem. 2014; 25:1438–1443. 25068829 10.1021/bc500302y

[26] Domnick C., Eggert F., Kath-Schorr S. Site-specific enzymatic introduction of a norbornene modified unnatural base into RNA and application in post-transcriptional labelling. Chem. Commun. 2015; 51:8253–8256. 10.1039/c5cc01765c 25874847

[27] Asare-Okai P.N., Agustin E., Fabris D., Royzen M. Site-specific fluorescence labelling of RNA using bio-orthogonal reaction of *trans*-cyclooctene and tetrazine. Chem. Commun. 2014; 50:7844–7847. 10.1039/c4cc02435d 24909672

[28] George J.T., Srivatsan S.G. Vinyluridine as a versatile chemoselective handle for the posttranscriptional chemical functionalization of RNA. Bioconjug. Chem. 2017; 28:1529–1536. 28406614 10.1021/acs.bioconjchem.7b00169 PMC6080697

[29] Agard N.J., Prescher J.A., Bertozzi C.R. A strain-promoted [3 + 2] azide-alkyne cycloaddition for covalent modification of biomolecules in living systems. J. Am. Chem. Soc. 2004; 126:15046–15047. 15547999 10.1021/ja044996f

[30] Rieder U., Luedtke N.W. Alkene–tetrazine ligation for imaging cellular DNA. Angew. Chem. Int. Ed. 2014; 53:9168–9172. 10.1002/anie.201403580 24981416

[31] Oliveira B.L., Guo Z., Bernardes G.J.L. Inverse electron demand Diels–Alder reactions in chemical biology. Chem. Soc. Rev. 2017; 46:4895–4950. 28660957 10.1039/c7cs00184c

[32] Spicer C.D., Davis B.G. Selective chemical protein modification. Nat. Commun. 2014; 5:4740. 25190082 10.1038/ncomms5740

[33] Yang M., Li J., Chen P.R. Transition metal-mediated bioorthogonal protein chemistry in living cells. Chem. Soc. Rev. 2014; 43:6511–6526. 24867400 10.1039/c4cs00117f

[34] Shaughnessy K.H. Palladium-catalyzed modification of unprotected nucleosides, nucleotides, and oligonucleotides. Molecules. 2015; 20:9419–9454. 26007192 10.3390/molecules20059419 PMC6272472

[35] Defrancq E., Messaoudi S. Palladium-mediated labeling of nucleic acids. Chembiochem. 2017; 18:426–431. 28000981 10.1002/cbic.201600599

[36] Jbara M., Maity S.K., Brik A. Palladium in the chemical synthesis and modification of proteins. Angew. Chem. Int. Ed. 2017; 56:10644–10655. 10.1002/anie.201702370 28383786

[37] Shaughnessy K.H. Hydrophilic ligands and their application in aqueous-phase metal-catalyzed reactions. Chem. Rev. 2009; 109:643–710. 19152291 10.1021/cr800403r

[38] Hervé G., Len C. Heck and Sonogashira couplings in aqueous media– application to unprotected nucleosides and nucleotides. Sustain. Chem. Process. 2015; 3:3. 10.3389/fchem.2015.00010 PMC4330919 25741506

[39] Ourailidou M.E., Dockerty P., Witte M., Poelarends G.J., Dekker F.J. Metabolic alkene labeling and *in vitro* detection of histone acylation *via* the aqueous oxidative Heck reaction. Org. Biomol. Chem. 2015; 13:3648–3653. 25672493 10.1039/c4ob02502d PMC4871226

[40] Omumi A., Beach D.G., Baker M., Gabryelski W., Manderville R.A. Postsynthetic guanine arylation of DNA by Suzuki−Miyaura cross-coupling. J. Am. Chem. Soc. 2011; 133:42–50. 21067186 10.1021/ja106158b

[41] Hollenstein M. Synthesis of deoxynucleoside triphosphates that include proline, urea, or sulfonamide groups and their polymerase incorporation into DNA. Chem. Eur. J. 2012; 18:13320–13330. 22996052 10.1002/chem.201201662

[42] Hocek M., Fojta M. Cross-coupling reactions of nucleoside triphosphates followed by polymerase incorporation. Construction and applications of base-functionalized nucleic acids. Org. Biomol. Chem. 2008; 6:2233–2241. 18563253 10.1039/b803664k

[43] Cahová H., Jäschke A. Nucleoside-based diarylethene photoswitches and their facile incorporation into photoswitchable DNA. Angew. Chem. Int. Ed. 2013; 52:3186–3190. 10.1002/anie.201209943 23404740

[44] Chalker J.M., Wood C.S.C., Davis B.G. A convenient catalyst for aqueous and protein Suzuki–Miyaura cross-coupling. J. Am. Chem. Soc. 2009; 131:16346–16347. 19852502 10.1021/ja907150m

[45] Spicer C.D., Triemer T., Davis B.G. Palladium-mediated cell-surface labeling. J. Am. Chem. Soc. 2012; 134:800–803. 22175226 10.1021/ja209352s

[46] Li N., Lim R.K.V., Edwardraja S., Lin Q. Copper-free Sonogashira cross-coupling for functionalization of alkyne-encoded proteins in aqueous medium and in bacterial cells. J. Am. Chem. Soc. 2011; 133:15316–15319. 21899368 10.1021/ja2066913 PMC3184007

[47] Lercher L., McGouran J.F., Kessler B.M., Schofield C.J., Davis B.G. DNA modification under mild conditions by Suzuki–Miyaura cross-coupling for the generation of functional probes. Angew. Chem. Int. Ed. 2013; 52:10553–10558. 10.1002/anie.201304038 PMC3823066 23943570

[48] Wicke L., Engels J.W. Postsynthetic on column RNA labeling via Stille coupling. Bioconjug. Chem. 2012; 23:627–642. 22321023 10.1021/bc200659j

[49] Jeong H.S., Hayashi G., Okamoto A. Diazirine photocrosslinking recruits activated FTO demethylase complexes for specific *N*^6^-methyladenosine recognition. ACS Chem. Biol. 2015; 10:1450–1455. 25751089 10.1021/cb5010096

[50] Wachowius F., Höbartner C. Chemical RNA modifications for studies of RNA structure and dynamics. Chembiochem. 2010; 11:469–480. 20135663 10.1002/cbic.200900697

[51] Xu W., Chan K.M., Kool E.T. Fluorescent nucleobases as tools for studying DNA and RNA. Nat. Chem. 2017; 9:1043–1055. 29064490 10.1038/nchem.2859 PMC5819341

[52] Sholokh M., Improta R., Mori M., Sharma R., Kenfack C., Shin D., Voltz K., Stote R.H., Zaporozhets O.A., Botta M. Tautomers of a fluorescent G surrogate and their distinct photophysics provide additional information channels. Angew. Chem. Int. Ed. 2016; 55:7974–7978. 10.1002/anie.201601688 PMC4978544 27273741

[53] Rovira A.R., Fin A., Tor Y. Expanding a fluorescent RNA alphabet: synthesis, photophysics and utility of isothiazole-derived purine nucleoside surrogates. Chem. Sci. 2017; 8:2983–2993. 28451365 10.1039/c6sc05354h PMC5380116

[54] Dumat B., Larsen A.F., Wilhelmsson L.M. Studying Z-DNA and B- to Z-DNA transitions using a cytosine analogue FRET-pair. Nucleic Acids Res. 2016; 44:e101. 26896804 10.1093/nar/gkw114 PMC4914084

[55] Nuthanakanti A., Boerneke M.A., Hermann T., Srivatsan S.G. Structure of the ribosomal RNA decoding site containing a selenium-modified responsive fluorescent ribonucleoside probe. Angew. Chem. Int. Ed. 2017; 56:2640–2644. 10.1002/anie.201611700 PMC5397316 28156044

[56] Puffer B., Kreutz C., Rieder U., Ebert M.-O., Konrat R., Micura R. 5-Fluoro pyrimidines: labels to probe DNA and RNA secondary structures by 1D ^19^F NMR spectroscopy. Nucleic Acids Res. 2009; 37:7728–7740. 19843610 10.1093/nar/gkp862 PMC2794194

[57] Olszewska A., Pohl R., Hocek M. Trifluoroacetophenone-linked nucleotides and DNA for studying of DNA–protein interactions by ^19^F NMR spectroscopy. J. Org. Chem. 2017; 82:11431–11439. 28991457 10.1021/acs.joc.7b01920

[58] Salon J., Jiang J., Sheng J., Gerlits O.O., Huang Z. Derivatization of DNAs with selenium at 6-position of guanine for function and crystal structure studies. Nucleic Acids Res. 2008; 36:7009–7018. 18986998 10.1093/nar/gkn843 PMC2602767

[59] Piton N., Mu Y., Stock G., Prisner T.F., Schiemann O., Engels J.W. Base-specific spin-labeling of RNA for structure determination. Nucleic Acids Res. 2007; 35:3128–3143. 17452362 10.1093/nar/gkm169 PMC1891445

[60] Shelke S.A., Sigurdsson S.T. Noncovalent and site-directed spin labeling of nucleic acids. Angew. Chem. Int. Ed. 2010; 49:7984–7986. 10.1002/anie.201002637 20845340

[61] Kerzhner M., Abdullin D., Więcek J., Matsuoka H., Hagelueken G., Schiemann O., Famulok M. Post-synthetic spin-labeling of RNA through click chemistry for PELDOR measurements. Chem. Eur. J. 2016; 22:12113–12121. 27412453 10.1002/chem.201601897

[62] Hocek M. Synthesis of base-modified 2′-deoxyribonucleoside triphosphates and their use in enzymatic synthesis of modified DNA for applications in bioanalysis and chemical biology. J. Org. Chem. 2014; 79:9914–9921. 25321948 10.1021/jo5020799

[63] Collier A., Wagner G. A facile two-step synthesis of 8-arylated guanosine mono- and triphosphates (8-aryl GXPs). Org. Biomol. Chem. 2006; 4:4526–4532. 17268649 10.1039/b614477b

[64] Čapek P., Cahová H., Pohl R., Hocek M., Gloeckner C., Marx A. An efficient method for the construction of functionalized DNA bearing amino acid groups through cross-coupling reactions of nucleoside triphosphates followed by primer extension or PCR. Chem. Eur. J. 2007; 13:6196–6203. 17487908 10.1002/chem.200700220

[65] Vaught J.D., Dewey T., Eaton B.E. T7 polymerase transcription with 5-position modified UTP derivatives. J. Am. Chem. Soc. 2004; 126:11231–11237. 15355104 10.1021/ja049009h

[66] Bley C.J., Qi X., Rand D.P., Borges C.R., Nelson R.W., Chen J.J.-L. RNA-protein binding interface in the telomerase ribonucleoprotein. Proc. Natl. Acad. Sci. U.S.A. 2011; 108:20333–20338. 22123986 10.1073/pnas.1100270108 PMC3251108

[67] Sinkeldam R.W., Greco N.J., Tor Y. Fluorescent analogs of biomolecular building blocks: design, properties, and applications. Chem. Rev. 2010; 110:2579–2619. 20205430 10.1021/cr900301e PMC2868948

[68] Tanpure A.A., Pawar M.G., Srivatsan S.G. Fluorescent nucleoside analogs: probes for investigating nucleic acid structure and function. Isr. J. Chem. 2013; 53:366–378.

[69] Mata G., Luedtke N.W. Fluorescent probe for proton-coupled DNA folding revealing slow exchange of i-motif and duplex structures. J. Am. Chem. Soc. 2015; 137:699–707. 25423623 10.1021/ja508741u

[70] Kanamori T., Ohzeki H., Masaki Y., Ohkubo A., Takahashi M., Tsuda K., Ito T., Shirouzu M., Kuwasako K., Muto Y. Controlling the fluorescence of benzofuran-modified uracil residues in oligonucleotides by triple-helix formation. Chembiochem. 2015; 16:167–176. 25469677 10.1002/cbic.201402346

[71] Dziuba D., Jurkiewicz P., Cebecauer M., Hof M., Hocek M. A rotational BODIPY nucleotide: an environment-sensitive fluorescence-lifetime probe for DNA interactions and applications in live-cell microscopy. Angew. Chem. Int. Ed. 2016; 55:174–178. 10.1002/anie.201507922 26768820

[72] Wranne M.S., Füchtbauer A.F., Dumat B., Bood M., El-Sagheer A.H., Brown T., Gradén H., Grøtli M., Wilhelmsson L.M. Toward complete sequence flexibility of nucleic acid base Analogue FRET. J. Am. Chem. Soc. 2017; 139:9271–9280. 28613885 10.1021/jacs.7b04517

[73] Manna S., Panse C.H., Sontakke V.A., Sangamesh S., Srivatsan S.G. Probing human telomeric DNA and RNA topology and ligand binding in a cellular model by using responsive fluorescent nucleoside probes. Chembiochem. 2017; 18:1604–1615.2017. 28569423 10.1002/cbic.201700283 PMC5724660

[74] Kimoto M., Mitsui T., Harada Y., Sato A., Yokoyama S., Hirao I. Fluorescent probing for RNA molecules by an unnatural base-pair system. Nucleic Acids Res. 2007; 35:5360–5369. 17693436 10.1093/nar/gkm508 PMC2018647

[75] Siraiwa S., Suzuki A., Katoh R., Saito Y. Design and synthesis of a novel fluorescent benzo[g]imidazo[4,5-c]quinoline nucleoside for monitoring base-pair-induced protonation with cytosine: distinguishing cytosine *via* changes in the intensity and wavelength of fluorescence. Org. Biomol. Chem. 2016; 14:3934–3942. 27044927 10.1039/c6ob00494f

[76] Srivatsan S.G., Tor Y. Fluorescent pyrimidine ribonucleotide: synthesis, enzymatic incorporation, and utilization. J. Am. Chem. Soc. 2007; 129:2044–2053. 17256858 10.1021/ja066455r PMC2517582

[77] Pawar M.G., Srivatsan S.G. Synthesis, photophysical characterization, and enzymatic incorporation of a microenvironment-sensitive fluorescent uridine analog. Org. Lett. 2011; 13:1114–1117. 21275418 10.1021/ol103147t

[78] Tanpure A.A., Srivatsan S.G. A microenvironment-sensitive fluorescent pyrimidine ribonucleoside analogue: synthesis, enzymatic incorporation, and fluorescence detection of a DNA abasic site. Chem. Eur. J. 2011; 17:12820–12827. 21956450 10.1002/chem.201101194

[79] Tanpure A.A., Srivatsan S.G. Conformation-sensitive nucleoside analogues as topology-specific fluorescence turn-on probes for DNA and RNA G-quadruplexes. Nucleic Acids Res. 2015; 43:e149. 26202965 10.1093/nar/gkv743 PMC4678839

[80] Sproviero M., Fadock K.L., Witham A.A., Manderville R.A. Positional impact of fluorescently modified G-tetrads within polymorphic human telomeric G-quadruplex structures. ACS Chem. Biol. 2015; 10:1311–1318. 25689465 10.1021/cb5009926

[81] Blanchard D.J.M., Cservenyi T.Z., Manderville R.A. Dual fluorescent deoxyguanosine mimics for FRET detection of G-quadruplex folding. Chem. Commun. 2015; 51:16829–16831. 10.1039/c5cc07154b 26435483

[82] Samanta B., Seikowski J., Höbartner C. Fluorogenic labeling of 5-formylpyrimidine nucleotides in DNA and RNA. Angew. Chem. Int. Ed. 2016; 55:1912–1916. 10.1002/anie.201508893 26679556

[83] Sinkeldam R.W., Greco N.J., Tor Y. Polarity of major grooves explored by using an isosteric emissive nucleoside. Chembiochem. 2008; 9:706–709. 18286575 10.1002/cbic.200700714

[84] Jadhav V.R., Barawkar D.A., Ganesh K.N. Polarity sensing by fluorescent oligonucleotides: first demonstration of sequence-dependent microenvironmental changes in the DNA major groove. J. Phys. Chem. B. 1999; 103:7383–7385.

[85] Doose S., Neuweiler H., Sauer M. Fluorescence quenching by photoinduced electron transfer: a reporter for conformational dynamics of macromolecules. Chemphyschem. 2009; 10:1389–1398. 19475638 10.1002/cphc.200900238

[86] Garrett C.E., Prasad K. The art of meeting palladium specifications in active pharmaceutical ingredients produced by Pd-catalyzed reactions. Adv. Synth. Catal. 2004; 346:889–900.

[87] Spicer C.D., Davis B.G. Palladium-mediated site-selective Suzuki–Miyaura protein modification at genetically encoded aryl halides. Chem. Commun. 2011; 47:1698–1700. 10.1039/c0cc04970k 21206952

