# Supplementary material for: Post-transcriptional labeling by using Suzuki–Miyaura cross-coupling generates functional RNA probes
Source: Nucleic Acids Res. 2018 Mar 13;46(11):e65. doi: 10.1093/nar/gky185 (PMC6009664; doi:10.1093/nar/gky185)
Supplement: Supplementary Data [file gky185_supplemental_files.doc]

Posttranscriptional Labeling by Using Suzuki–Miyaura Cross-Coupling Generates Functional RNA Probes

Manisha B. Walunj, Arun A. Tanpure and Seergazhi G. Srivatsan*

**Supplementary Data**

| **Content** | Page |
| --- | --- |
| 1. Materials | S2 |
| 2. Instrumentation | S2 |
| 3. Synthesis  5-iodouridine 5′-triphosphate (IUTP **2**)  Synthesis of amine-containing pinacol boronate ester linkers **21** and **22**  Synthesis of NBD- and biotin-tagged pinacol boronate esters (**9**‒**11**)  Synthesis of 2-vinylbenzothiophene- and 2-vinylbenzofuran-boronic esters **16** and **17** | S2-S5 |
| **Supplementary Figure S1.** Phosphor image of transcripts obtained by transcription of templates **T1**–**T5** in the presence of UTP and or IUTP **2** | S5 |
| 4. General procedure for the sample preparation for MALDI-TOF mass analysis | S6 |
| **Supplementary Figure S2.** HPLC chromatogram and MALDI-TOF mass spectrum of IU-labeled RNA transcript **4** | S6 |
| **Supplementary Figure S3.** RP-HPLC chromatogram and MALDI-TOF mass spectrum of coupled RNA ON products **9a**, **10a** and **11a** | S7 |
| **Supplementary Table S1.** Mass data of Suzuki–Miyaura cross-coupled RNA ON products | S8 |
| **Supplementary Figure S4.** RP-HPLC chromatogram and MALDI-TOF mass spectrum of coupled RNA ON products **12a**, **13a**, **14a** and **15a** | S9 |
| **Supplementary Figure S5.** RP-HPLC chromatogram of reaction mixture of Suzuki–Miyaura cross-coupling between IU-labeled transcript **4** and boronic ester **16** and **17** using L1 and L2 | S10 |
| **Supplementary Figure S6.** MALDI-TOF mass spectrum of coupled RNA ON products **16aʹ**, **17aʹ**, **19aʹ** and **20aʹ**. | S11 |
| **Supplementary Figure S7.** RP-HPLC profile of ribonucleoside products obtained after enzymatic digestion of Suzuki-coupled RNA ON **17aʹ** | S12 |
| **Supplementary Table S2.** MALDI-TOF mass analysis of HPLC fractions of RNA ON **17aʹ** digest | S12 |
| Synthesis of *trans* form of 5-(benzofuran-2-yl)vinyl uridine (**18**) | S13 |
| **Supplementary Figure S8.** Control Suzuki coupling reactions with IU-labeled RNA ON **4** and unmodified RNA ON **3** | S14 |
| **Supplementary Figure S9.** Absorption and emission spectra of 5-(benzofuran-2-yl)vinyl uridine **18** in solvents of different polarity | S15 |
| **Supplementary Table S3.** Photophysical properties of modified nucleoside **18** in different solvents. | S15 |
| 5. NMR spectra | S16 |
| 6. References | S23 |

**1. Materials:** All chemicals and biochemicals purchased were used as supplied unless otherwise stated. 5-iodouridine (IU **1**) was prepared according to a literature report (1). Boronic acids **12** and **15** were purchased from Sigma-Aldrich, whereas **13** and **14** were purchased from Alfa Aesar. 4-Chloro-7-nitrobenzofurazan, biotin, bis(pinacolato)diboron, 2-aminopyrimidine-4,6-diol (ADHP, L1) were purchased from Sigma-Aldrich. 2-(dimethylamino)pyrimidine-4,6-diol (DMADHP, L2) was prepared according to a literature report (2). T7 RNA polymerase, ribonuclease inhibitor (RiboLock), NTPs, RNase A and RNase T1 were obtained from Fermentas, Thermo Fisher Scientific. DNA oligonucleotides (ONs) purchased from Integrated DNA Technologies, Inc., were purified by gel electrophoresis under denaturing condition and desalted using Sep-Pak Classic C18 cartridges (Waters Corporation). Calf intestinal alkaline phosphatase (CIP) and snake venom phosphodiesterase I were procured from Invitrogen and Sigma-Aldrich, respectively. POCl_3_ was purchased from Across Organics and freshly distilled prior to use. Radiolabeled α-^32^P ATP (2000 Ci/mmol) was purchased from the Board of Radiation and Isotope Technology, Government of India. Chemicals for preparing buffer solutions were purchased from Sigma-Aldrich (HPLC or BioUltra grade). Autoclaved water was used in all biochemical reactions.

**2. Instrumentation**: NMR spectra were recorded on a 400 MHz Jeol ECS-400 and Bruker Avance III HD Ascend 400 MHz spectrometer. Mass measurements were recorded on Applied Biosystems 4800 Plus MALDI TOF/TOF analyzer and Bruker Avance III HD Ascend 400 MHz mass spectrometer. Steady-state fluorescence experiments were carried out in a micro fluorescence cuvette (Hellma, path length 1.0 cm) on a Horiba JobinYvon, Fluorolog-3. Reversed-phase (RP) flash chromatographic (C18 Redi*SepRf* column) purifications were carried out using Teledyne ISCO, Combi Flash *Rf*. HPLC analysis was performed using Agilent Technologies 1260 Infinity.

**3. Synthesis**


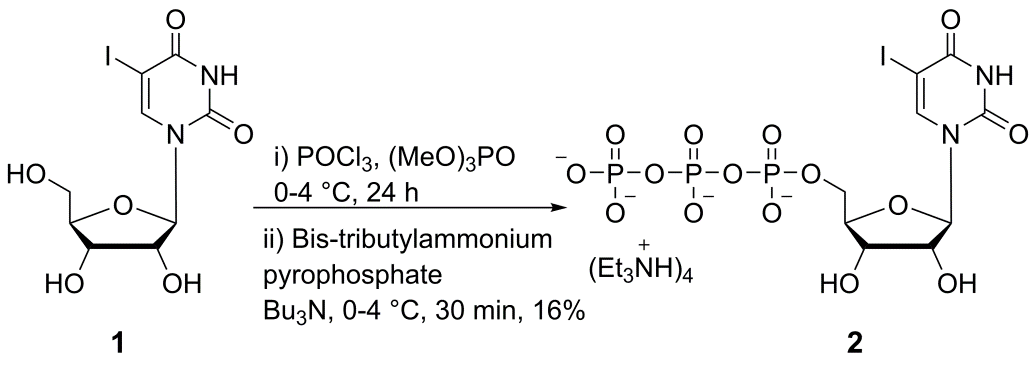


**5-iodouridine 5′-triphosphate (IUTP 2):** To an ice cold solution of 5-iodouridine **1** (96 mg, 0.26 mmol, 1 equiv.) in trimethyl phosphate (1.20 mL) was added freshly distilled POCl_3_ (61 µL, 0.65 mmol, 2.5 equiv.). The solution was stirred for 24 at ~4 °C. TLC revealed only partial conversion of the ribonucleoside into the product. *Bis-*tributylammonium pyrophosphate (3, 0.5 M in DMF, 2.7 mL, 1.35 mmol, 5.2 equiv.) and tributylamine (670 µL, 2.86 mmol, 11 equiv.) were rapidly added simultaneously under ice-cold conditions. The reaction was quenched after 30 min with 1 M triethylammonium bicarbonate buffer (TEAB, pH 7.5), and was extracted with distilled ethyl acetate. The aqueous layer was evaporated under vacuum and the residue was purified first on a DEAE sephadex-A25 anion exchange column (10 mM–1 M TEAB buffer, pH 7.5) followed by reversed-phase flash column chromatography (C18 Redi*SepRf*, 0–50% acetonitrile in 50 mM triethylammonium acetate buffer, pH 7.3, 45 min). Appropriate fractions were lyophilized to afford IUTP **2** as a tetratriethylammonium salt (61 mg, 16%); ^1^H NMR (400 MHz, D_2_O): δ (ppm) 8.24 (s, 1H), 5.89 (d, *J* = 5.2 Hz, 1H), 4.42–4.35 (m, 2H), 4.23–4.16 (m, 3H); ^31^P NMR (162 MHz, D_2_O): δ (ppm) -7.75 (br, P_γ_), -11.10 (d, *J* = 18.31, P_α_), -22.06 (br, P_β_); HRMS: m/z Calcd. for C_9_H_13_IN_2_O_15_P_3_ [M-H]^-^ = 608.8573, found = 608.8574. Analytical data matches with the literature report (4).

**Synthesis of amine-containing pinacol boronate ester linkers 21 and 22: 21** and **22** were prepared by following a literature procedure (5). **21** and **22** were characterized by ^1^H NMR, ^13^C NMR and HRMS, which were consistent with reported data.


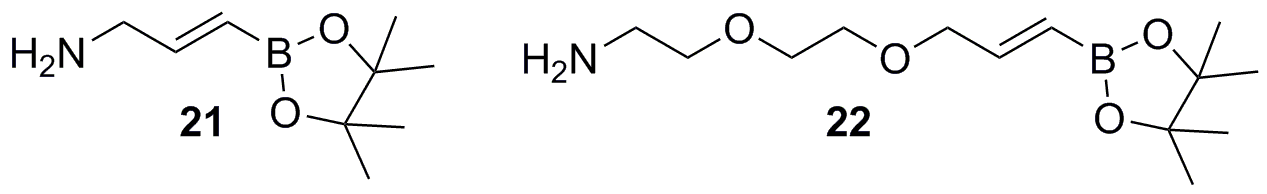


**Synthesis of NBD- and biotin-tagged pinacol boronate esters (9**‒**11)**

**(E)-7-nitro-N-(3-(4,4,5,5-tetramethyl-1,3,2-dioxaborolan-2-yl) allyl)benzo[c][1,2,5] oxadiazol-4-amine (9):**

**
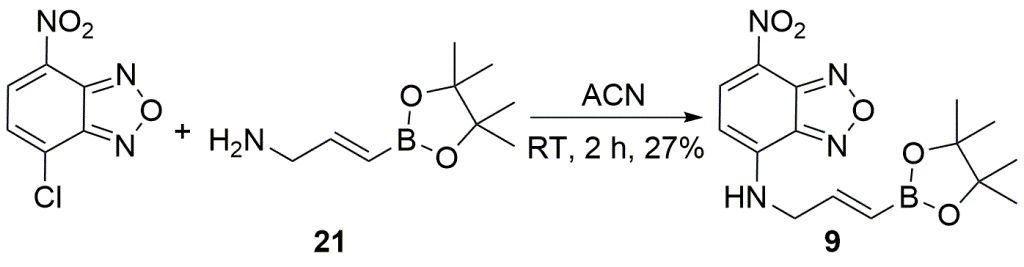
**

4-Chloro-7-nitrobenzofurazan (95 mg, 0.48 mmol, 1 equiv.) was dissolved in acetonitrile (4 mL). A solution of amine boronic ester **21** (176 mg, 0.96 mmol, 2 equiv.) in acetonitrile (1 mL) was added dropwise. Reaction mixture was stirred at RT for 2 h. Solvent was evaporated and the residue was purified by silica gel column chromatography to afford NBD boronic ester **9** as an orange-brown solid (45 mg, 27%). TLC (EtOAc:pet ether = 30:70); *R_f_* = 0.60. ^1^H NMR (400 MHz, CDCl_3_ containing 0.03% (v/v) TMS) δ (ppm) 8.48 (d, *J* = 8.6 Hz, 1H), 6.65 (dt, *J* = 18, 4.6 Hz, 1H), 6.46 (br, 1H), 6.16 (d, *J* = 8.6 Hz, 1H), 5.75 (dt, *J* = 18, 1.6 Hz, 1H), 4.25–4.22 (m, 2H), 1.27 (s, 12H); ^13^C NMR (100 MHz, CDCl_3_ containing 0.03% (v/v) TMS) δ (ppm) 144.31, 144.27, 143.80, 143.43, 136.27, 124.63, 99.39, 83.79, 47.03, 24.78; HRMS: m/z Calcd. for C_15_H_20_BN_4_O_5_ [M+H]^+^ = 347.1527, found = 347.1526.

**(E)-7-nitro-N-(2-((3-(4,4,5,5-tetramethyl-1,3,2-dioxaborolan-2-yl)allyl)oxy)ethyl)benzo[c] [1,2,5]oxadiazol-4-amine (10):**


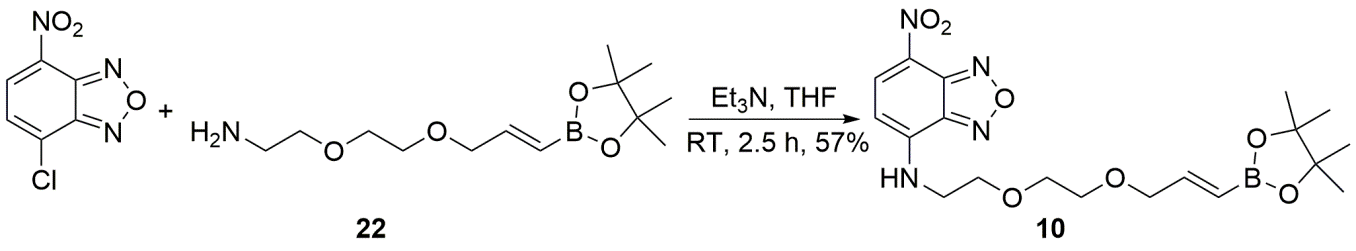


A solution of amine boronic ester **22** (191 mg, 0.70 mmol, 1 equiv.) in anhydrous THF (2 mL) was treated with Et_3_N (195 µL, 1.40 mmol, 2 equiv.). To this reaction mixture, a solution of 4-chloro-7-nitro benzofurazan (183 mg, 0.91 mmol, 1.3 equiv.) in anhydrous THF (1 mL) was added dropwise. The resulting mixture was stirred at RT for 2.5 h. After completion, the reaction mixture was diluted with ethyl acetate and washed with saturated aq. NH_4_Cl, saturated aq. NaHCO_3_ and brine. The organic layer was dried over Na_2_SO_4_ and filtered. The resulting filtrate was evaporated and the obtained residue was purified by silica gel column chromatography to afford the pure product **10** as dark orange solid (175 mg, 57%). TLC (EtOAc:pet ether = 60:40); *R_f_* = 0.67; ^1^H NMR (400 MHz, CDCl_3_ containing 0.03% (v/v) TMS) δ (ppm) 8.49 (d, *J* = 8.6 Hz, 1H), 6.99 (br, 1H), 6.64 (dt, *J* = 18.4, 4.6 Hz, 1H), 6.19 (d, *J* = 8.6 Hz, 1H), 5.71 (dt, *J* = 18.4, 1.6 Hz, 1H), 4.16 (dd, *J* = 4.6, 1.6 Hz, 2H), 3.88 (t, *J* = 5.2 Hz, 2H), 3.75–3.73 (m, 2H), 3.71–3.65 (m, 4H), 1.26 (s, 12H); ^13^C NMR (100 MHz, CDCl_3_ containing 0.03% (v/v) TMS) δ (ppm) 148.6, 144.3, 144.2, 143.9, 136.4, 123.9, 98.8, 83.4, 72.8, 70.7, 69.9, 68.3, 43.7, 24.8; HRMS: m/z Calcd. for C_19_H_28_BN_4_O_7_ [M+H]^+^ = 435.2051, found = 435.2069.

**5-((4S)-2-oxohexahydro-1H-thieno[3,4-d]imidazol-4-yl)-N-(2-(((E)-3-(4,4,5,5-tetramethyl -1,3,2-dioxaborolan-2-yl)allyl)oxy)ethyl)pentanamide (11):**

**
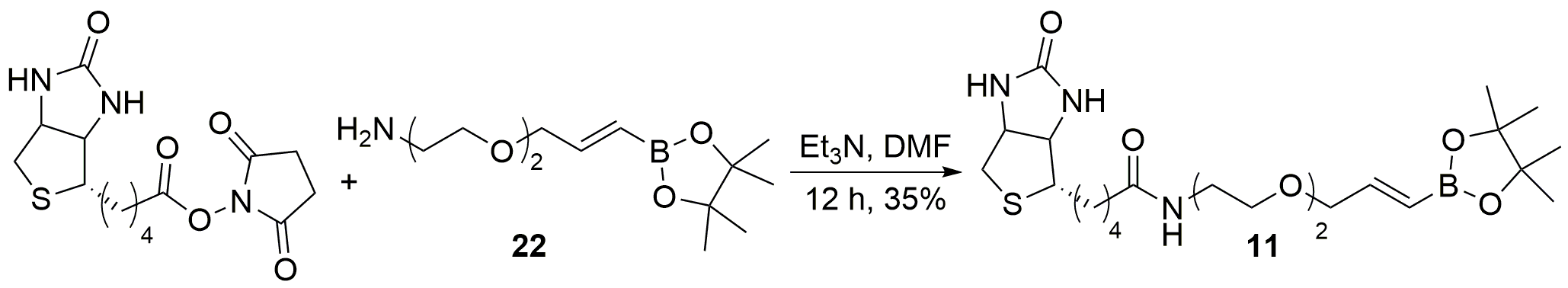
**

NHS-ester of biotin (6, 200 mg, 0.59 mmol, 1 equiv.) was dissolved in 3 mL of anhydrous DMF followed by addition of dry Et_3_N (165 µL, 1.18 mmol, 2 equiv.). A solution of amine boronic ester **22** (208 mg, 0.77 mmol, 1.3 equiv.) in 2 mL dry DMF was added, and the reaction mixture was stirred at RT for 12 h. Solvent was evaporated and the residue was purified by silica gel column chromatography to afford the product **11** as a white solid (105 mg, 35%). TLC (MeOH:CH_2_Cl_2_ = 10:90); *R_f_* = 0.60; ^1^H NMR (400 MHz, CDCl_3_ containing 0.03% (v/v) TMS) δ (ppm) 6.78 (t, *J* = 5.4 Hz, 1H), 6.62 (dt, *J* = 18, 4.6 Hz, 1H), 6.56 (s, 1H), 5.70 (dt, *J* = 18, 1.8 Hz, 1H), 5.62 (s, 1H), 4.52–4.48 (m, 1H), 4.33–4.10 (m, 1H), 4.10 (dd, *J* = 4.6, 1.8 Hz, 2H), 3.64–3.59 (m, 4H), 3.57 (t, *J* = 5 Hz, 2H), 3.49–3.39 (m, 2H), 3.16–3.11 (m, 1H), 2.90 (dd, *J* = 12.8, 4.8 Hz, 1H), 2.74 (d, *J* = 12.8 Hz, 1H), 2.27–2.20 (m, 2H), 1.81–1.61 (m, 4H), 1.48–1.40 (m, 2H), 1.27 (s, 12H); ^13^C NMR (100 MHz, CDCl_3_ containing 0.03% (v/v) TMS) δ (ppm) 173.41, 164.09, 148.82, 83.38, 72.70, 70.18, 70.00, 69.57 , 61.74, 60.23, 55.54, 40.55, 39.15, 35.95, 28.14, 28.05, 25.57, 24.79; HRMS: m/z Calcd. for C_23_H_41_BN_3_O_6_S [M+H]^+^ = 498.2809, found = 498.2814.

**Synthesis of 2-vinylbenzothiophene- and 2-vinylbenzofuran-boronic esters 16 and 17:**


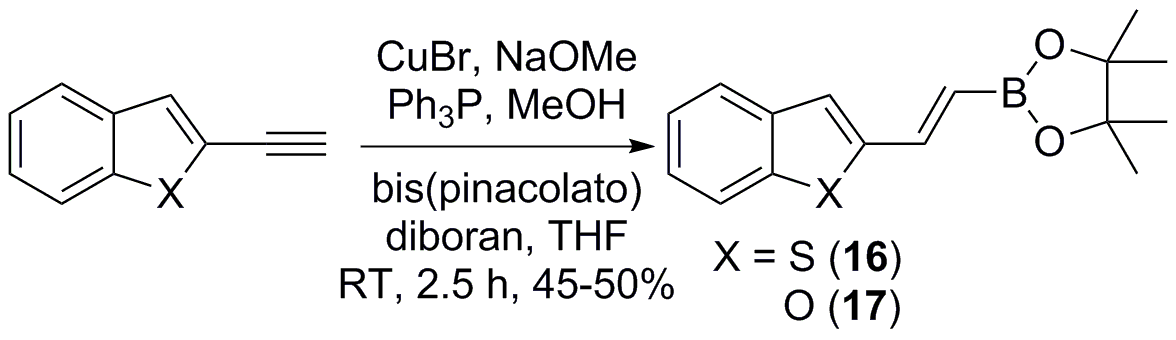


**General procedure**: A mixture of bis(pinacolato)diboron (1.1 equiv.), sodium methoxide (0.20 equiv.), triphenylphosphine (6 mol%) and copper bromide (5 mol%) were stirred under nitrogen for 30 min at RT in dry THF. To this mixture, 2-ethynylbenzothiophene/2-ethynylbenzofuran (1 equiv., 7,8) in methanol (2 equiv) was added. The reaction mixture was stirred for another 2 h at RT. After completion of reaction, solvent was evaporated. To the obtained residue, distilled hexane was added and filtered through celite pad. The filtrate was concentrated under reduced pressure. The residue was purified by silica gel column chromatography to afford the desired product as a yellow solid.

**(E)-2-(2-(benzo[b]thiophen-2-yl)vinyl)-4,4,5,5-tetramethyl-1,3,2-dioxaborolane (16):**

A mixture of 2-ethynylbenzothiophene (200 mg, 1.26 mmol, 1 equiv.), bis(pinacolato)diboron (352 mg, 1.39 mmol, 1.1 equiv.), sodium methoxide (14 mg, 0.25 mmol, 0.20 equiv.), triphenylphosphine (20 mg, 0.076 mmol, 6 mol%) and copper bromide (9 mg, 0.06 mmol, 5 mol%) yielded **16** (160 mg, 45%). TLC (EtOAc:pet ether = 10:90); *R_f_* = 0.37; ^1^H NMR (400 MHz, CDCl_3_) δ (ppm) 7.78–7.75 (m, 1H), 7.74–7.69 (m, 1H), 7.56 (d, *J =* 18 Hz, 1H), 7.33–7.29 (m, 2H), 7.26 (s, 1H), 6.01 (d, *J =* 18.0 Hz, 1H), 1.32 (s, 12H); ^13^C NMR (100 MHz, CDCl_3_) δ (ppm) 144.1, 142.5, 140.1, 139.8, 125.4, 125.2, 124.6, 124.2, 122.5, 83.7, 25.0; HRMS: m/z Calcd. for C_16_H_20_BO_2_S [M+H]^+^ = 287.1277, found = 287.1278.

**(E)-2-(2-(benzofuran-2-yl)vinyl)-4,4,5,5-tetramethyl-1,3,2-dioxaborolane (17):**

A mixture of 2-ethynylbenzofuran (283 mg, 1.99 mmol, 1 equiv.), bis(pinacolato)diboron (559 mg, 2.2 mmol, 1.1 equiv.), sodium methoxide (22 mg, 0.40 mmol, 0.20 equiv.), triphenylphosphine (32 mg, 0.12 mmol, 6 mol%) and copper bromide (14 mg, 0.10 mmol, 5 mol%) yielded **17** (270 mg, 50%). TLC (EtOAc:pet ether = 10:90); *R_f_*  = 0.50; ^1^H NMR (400 MHz, CDCl_3_) δ (ppm) 7.55–7.53 (m, 1H), 7.47–7.45 (m, 1H), 7.31–7.25 (m, 2H), 7.22–7.18 (m, 1H), 6.72 (s, 1H), 6.32 (d, *J =* 18.0 Hz, 1H), 1.32 (s, 12H); ^13^C NMR (100 MHz, CDCl_3_) δ (ppm) 155.3, 155.1, 136.6, 128.9, 125.4, 123.0, 121.5, 111.4, 107.3, 83.7, 24.9; HRMS: m/z Calcd. for C_16_H_20_BO_3_ [M+H]^+^ = 271.1505, found = 271.1509.

**
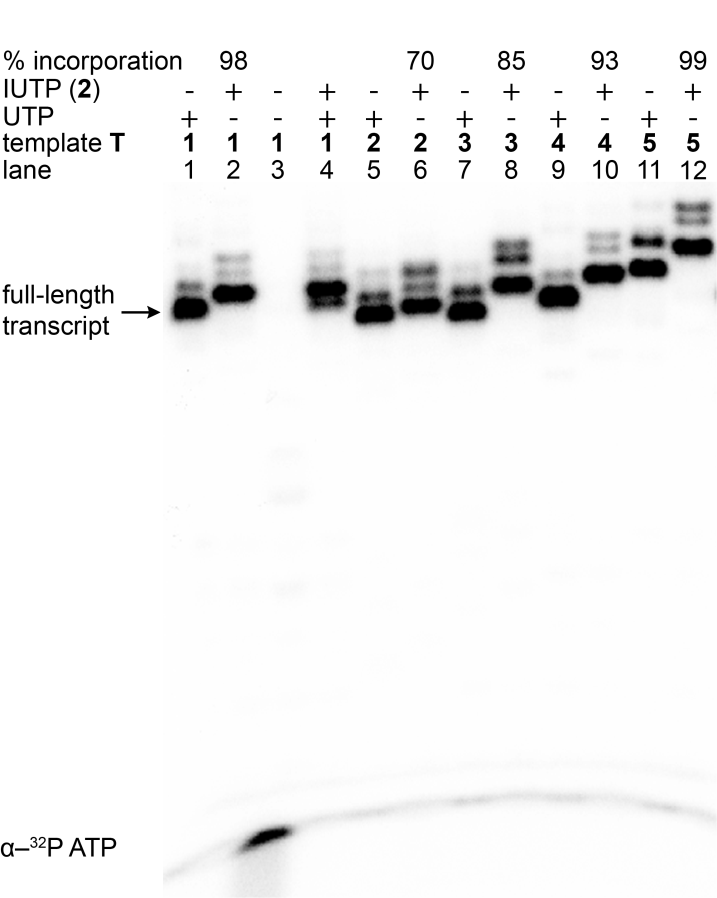
**

**Supplementary Figure S1.** Phosphor image of transcripts obtained by transcription of templates **T1**–**T5** in the presence of UTP and or IUTP **2**. % Incorporation of **2** is reported relative to a control transcription with UTP.

**4**. **General procedure for the sample preparation for MALDI-TOF mass analysis of transcript and posttranscriptionally coupled RNA ON products:** Sample for mass analysis was prepared by combining 1 µL of the transcript/coupled RNA product (~200 µM), 2.5 µL of DNA standard (100 µM, 18-mer) and 5 μL of a mixture of saturated 3-hydroxy picolinic acid and 100 mM ammonium citrate buffer (pH 9, in the ratio of 9:1). The sample was desalted using ion-exchange resin (Dowex 50W-X8, 100-200 mesh, ammonium cation form), spotted on the MALDI plate, and was air dried. The resulting spectrum was calibrated relative to an internal 18-mer DNA ON standard (See Figure S2 and Table S1). Depending on the peak intensity the ratio of RNA ON and internal DNA standard was varied.


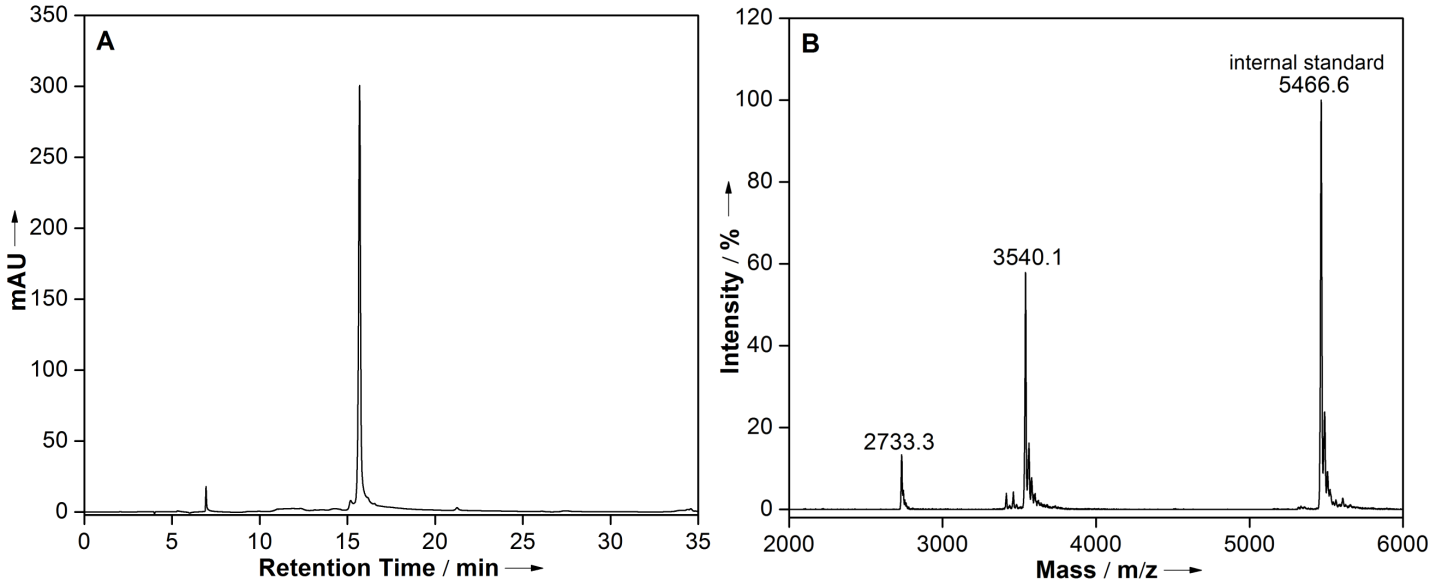


**Supplementary Figure S2.** (**A**) HPLC chromatogram of PAGE purified IU-labeled RNA transcript **4** at 260 nm. Mobile phase A = 50 mM triethylammonium acetate buffer (TEAA, pH 7.0), mobile phase B = acetonitrile. Flow rate = 1 mL/min. Gradient = 0−30% B in 35 min, 30−100% B in 10 min and 100% B for 5 min. HPLC analysis was performed using Phenomenex-Luna C18 column (250 x 4.6 mm, 5 micron).

(**B**) MALDI-TOF mass spectrum of RNA ON **4**. Spectrum is calibrated with respect to the +1 and +2 ion of an internal 18-mer DNA ON standard (m/z for +1 and +2 ion are 5466.6 and 2733.3 respectively). Calcd. mass for IU-modified RNA transcript **4**: [M]^+^ 3540.8; found: [M]^+^ 3540.1.

**
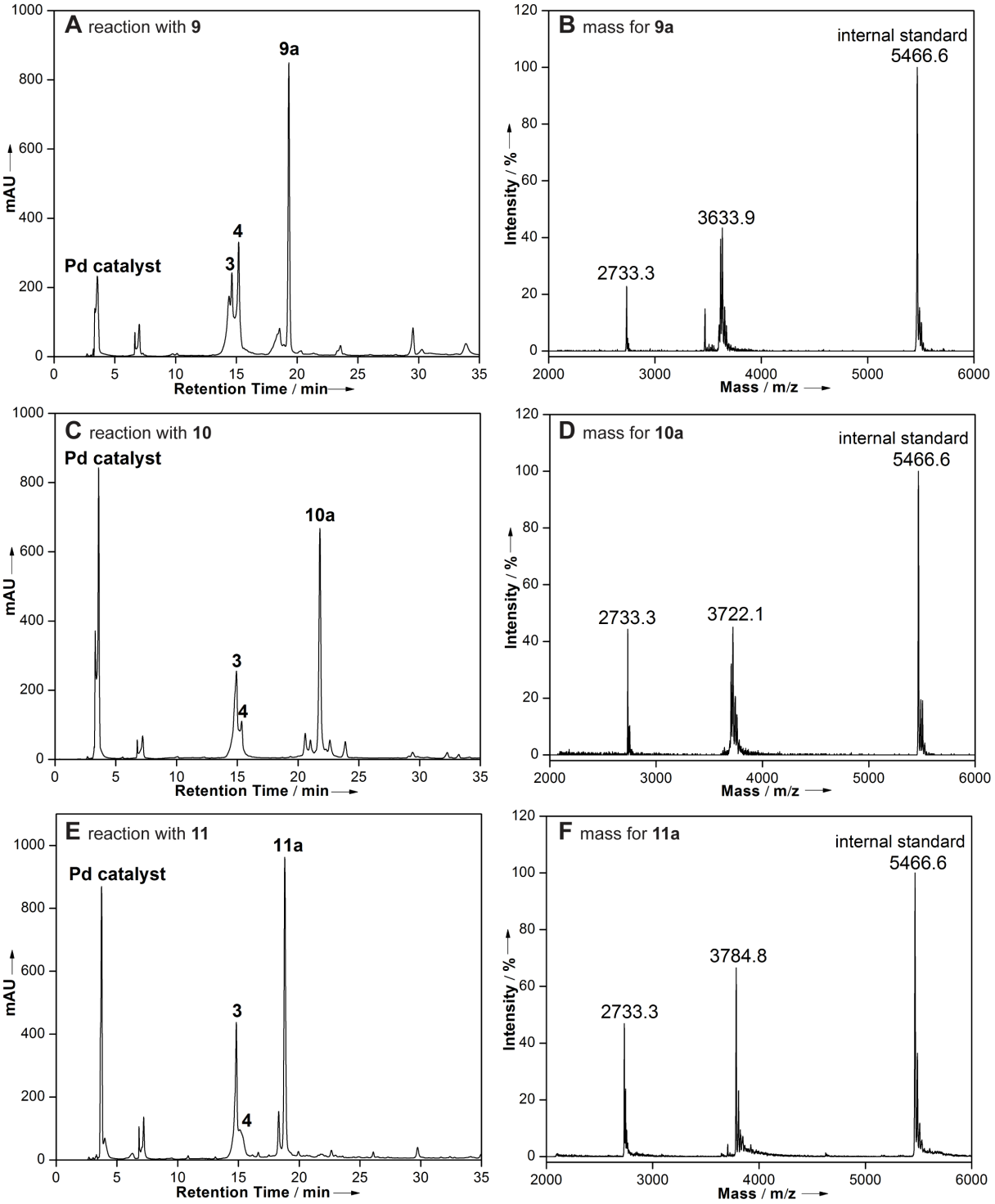
**

**Supplementary Figure S3.** (**A**, **C**, **E**) RP-HPLC chromatogram of reaction mixture of Suzuki–Miyaura cross-coupling between IU-labeled transcript **4** and boronic esters **9**–**11**. **9a**−**11a** correspond to coupled RNA ON product; **3** is deiodinated transcript. Mobile phase A: 50 mM TEAA buffer (pH 7.0); mobile phase B: acetonitrile. Flow rate: 1 mL/min. Gradient: 0−30% B in 35 min, 30−100% B in 10 min and 100% B for 5 min.

(**B**, **D**, **F**) MALDI-TOF mass spectrum of coupled RNA ON products **9a**, **10a** and **11a** (HPLC fractions) obtained from reactions between transcript **4** and boronic esters **9**–**11**, respectively. See Figure 5 for the structure of the product. Spectrum is calibrated with respect to the +1 and +2 ion of an internal 18-mer DNA ON standard (m/z for +1 and +2 ion are 5466.6 and 2733.3 respectively). See Table 1 and Table S1 for isolated yield and mass data.

**Supplementary Table S1.** Mass data of Suzuki–Miyaura cross-coupled RNA ON products obtained by posttranscriptional chemical modification of IU-labeled RNA ON transcripts.

| Entry | Cross-coupled RNA ON product | MALDI-TOF analysis of product (m/z) [M]^+^ | |
| --- | --- | --- | --- |
|  |  | Calculated | Observed |
| 1 | **9a** | 3633.1 | 3633.9 |
| 2 | **10a** | 3721.2 | 3722.1 |
| 3 | **11a** | 3784.4 | 3784.8 |
| 4 | **12a** | 3481.0 | 3482.0 |
| 5 | **13a** | 3547.1 | 3546.6 |
| 6 | **14a** | 3531.0 | 3531.0 |
| 7 | **15a** | 3481.0 | 3481.1 |
| 8 | **16aʹ** | 3573.1 | 3573.3 |
| 9 | **17aʹ** | 3557.1 | 3556.8 |
| 10 | **19aʹ** | 3477.0 | 3476.4 |
| 11 | **20aʹ** | 3525.1 | 3524.6 |


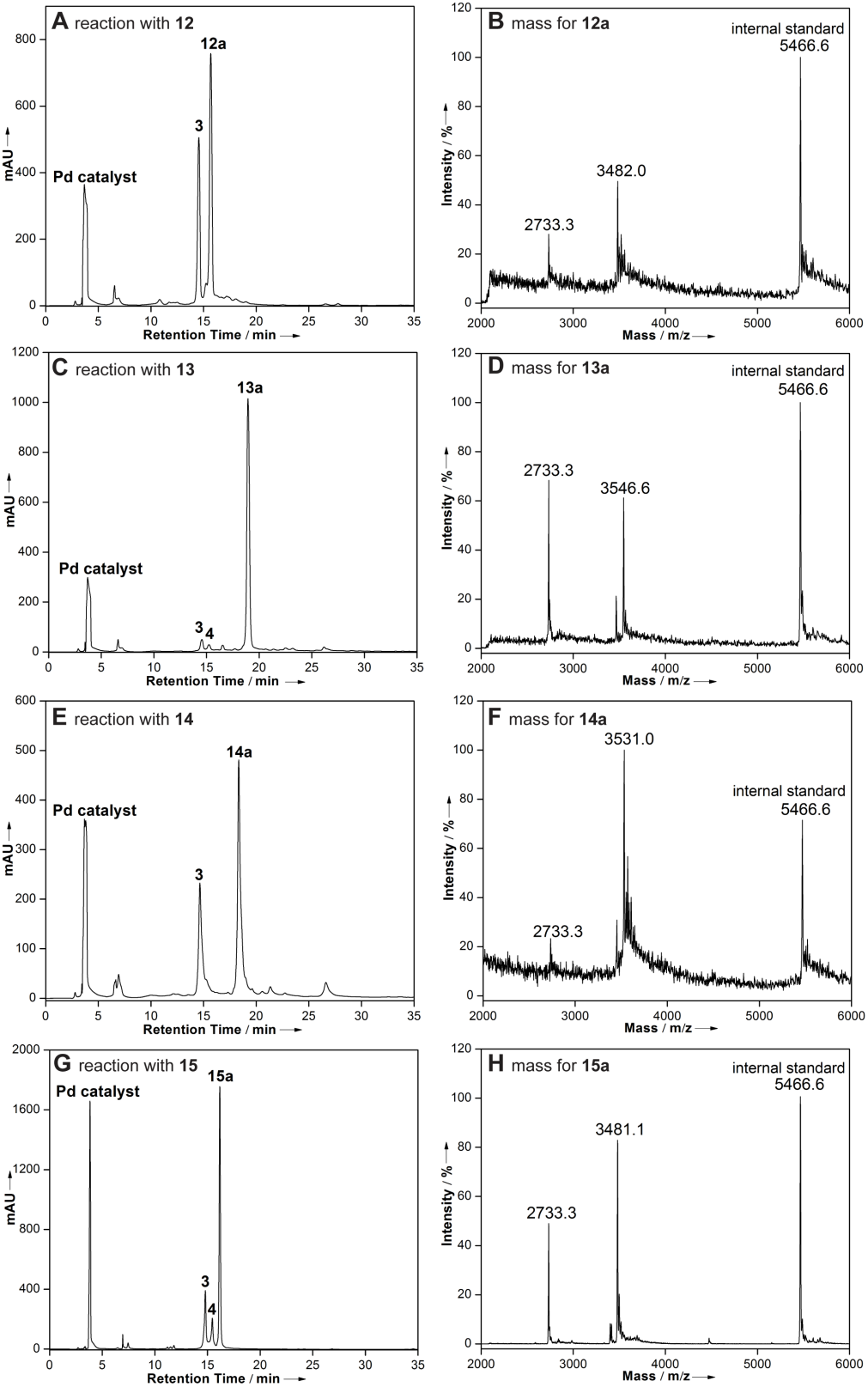


**Supplementary Figure S4.** (**A**, **C**, **E**, **G**) RP-HPLC chromatogram of reaction mixture of Suzuki–Miyaura cross-coupling between IU-labeled transcript **4** and boronic acids/ester **12**–**15**. **12a**−**15a** correspond to coupled RNA ON product; **3** is deiodinated transcript. Mobile phase A: 50 mM TEAA buffer (pH 7.0); mobile phase B: acetonitrile. Flow rate: 1 mL/min. Gradient: 0−30% B in 35 min, 30−100% B in 10 min and 100% B for 5 min.

(**B**, **D**, **F, H**) MALDI-TOF mass spectrum of coupled RNA ON products **12a**, **13a**, **14a** and **15a** (HPLC fractions) obtained from reactions between transcript **4** and boronic acids/ester **12**–**15**, respectively. See Figure 5 for the structure of the product. Spectrum is calibrated with respect to the +1 and +2 ion of an internal 18-mer DNA ON standard (m/z for +1 and +2 ion are 5466.6 and 2733.3 respectively). See Table 1 and Table S1 for isolated yield and mass data.


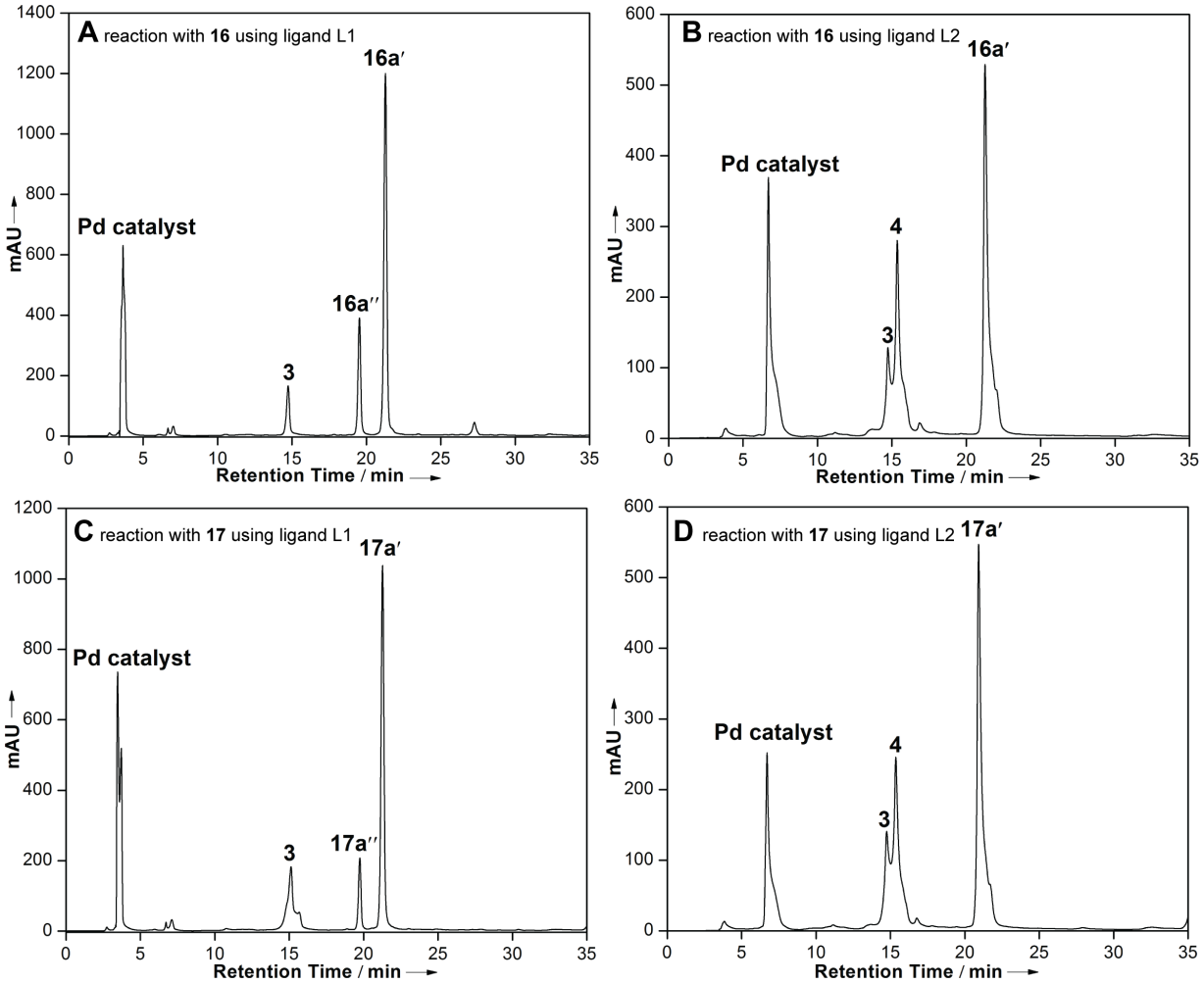


**Supplementary Figure S5.** RP-HPLC chromatogram of reaction mixture of Suzuki–Miyaura cross-coupling between IU-labeled transcript **4** and boronic ester **16** and **17**. **A** and **B**: reaction with boronic ester **16** using ligand L1 and L2, respectively. **C** and **D**: reaction with **17** using ligand L1 and L2, respectively.

While major peaks **16aʹ** and **17aʹ** correspond to the “*trans”* isomer product, minor peaks **16aʹʹ** and **17aʹʹ** correspond to the *“cis”* isomer product. See Table S1 for mass data.

Importantly, reaction in the presence of Pd-L2 catalytic system yielded only the *trans* products **16aʹ** and **17aʹ**, suggesting that this posttranscriptional modification is an example of a ligand-controlled stereoselective alkenylation process.

Mobile phase A: 50 mM TEAA buffer (pH 7.0), mobile phase B: acetonitrile. Flow rate: 1 mL/min. Gradient: 0−30 % B in 35 min, 30−100% B in 10 min and 100% B for 5 min.


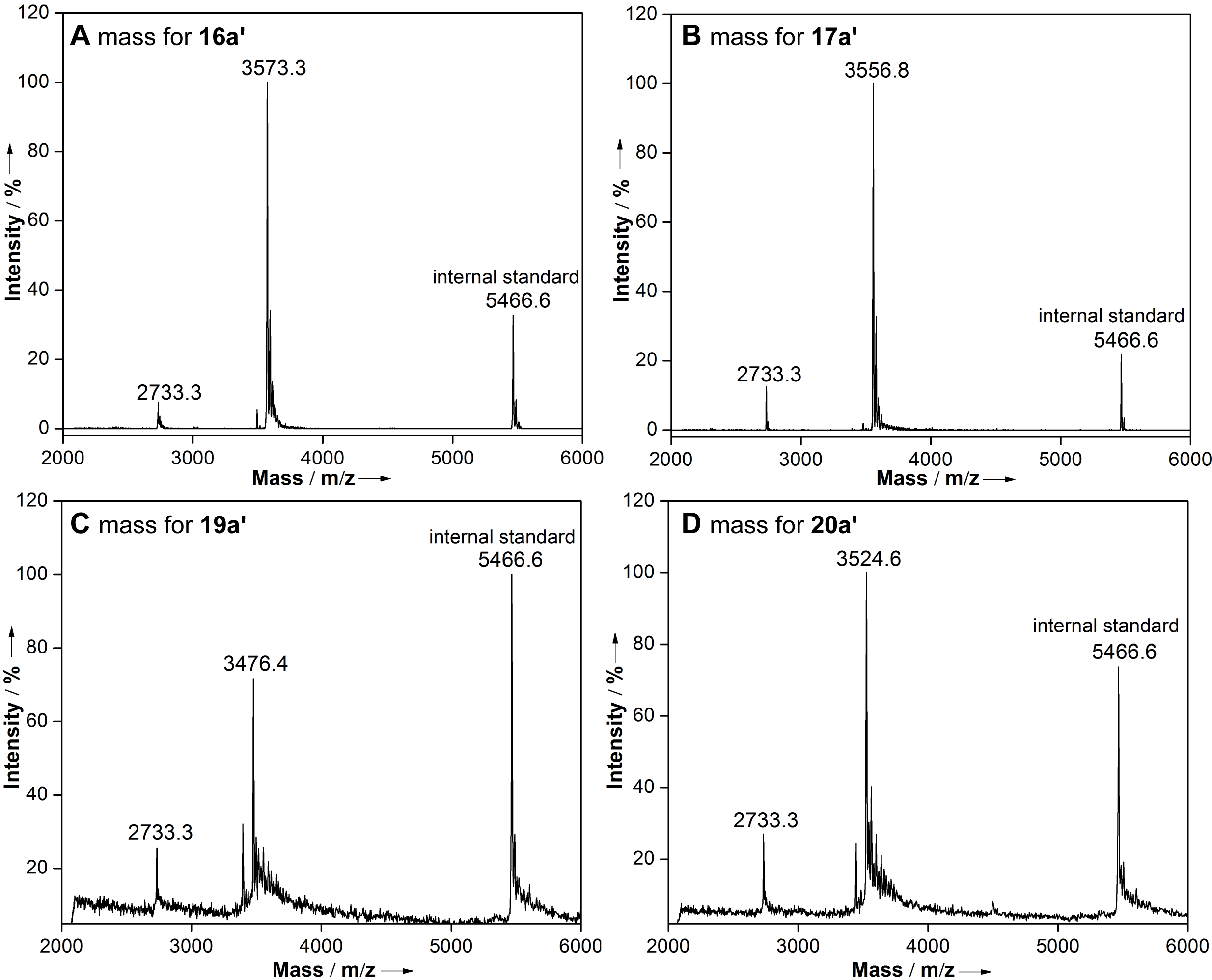


**Supplementary Figure S6.** MALDI-TOF mass spectrum of coupled RNA ON products **16aʹ**, **17aʹ**, **19aʹ** and **20aʹ**. For details see section 4 and Table S1.


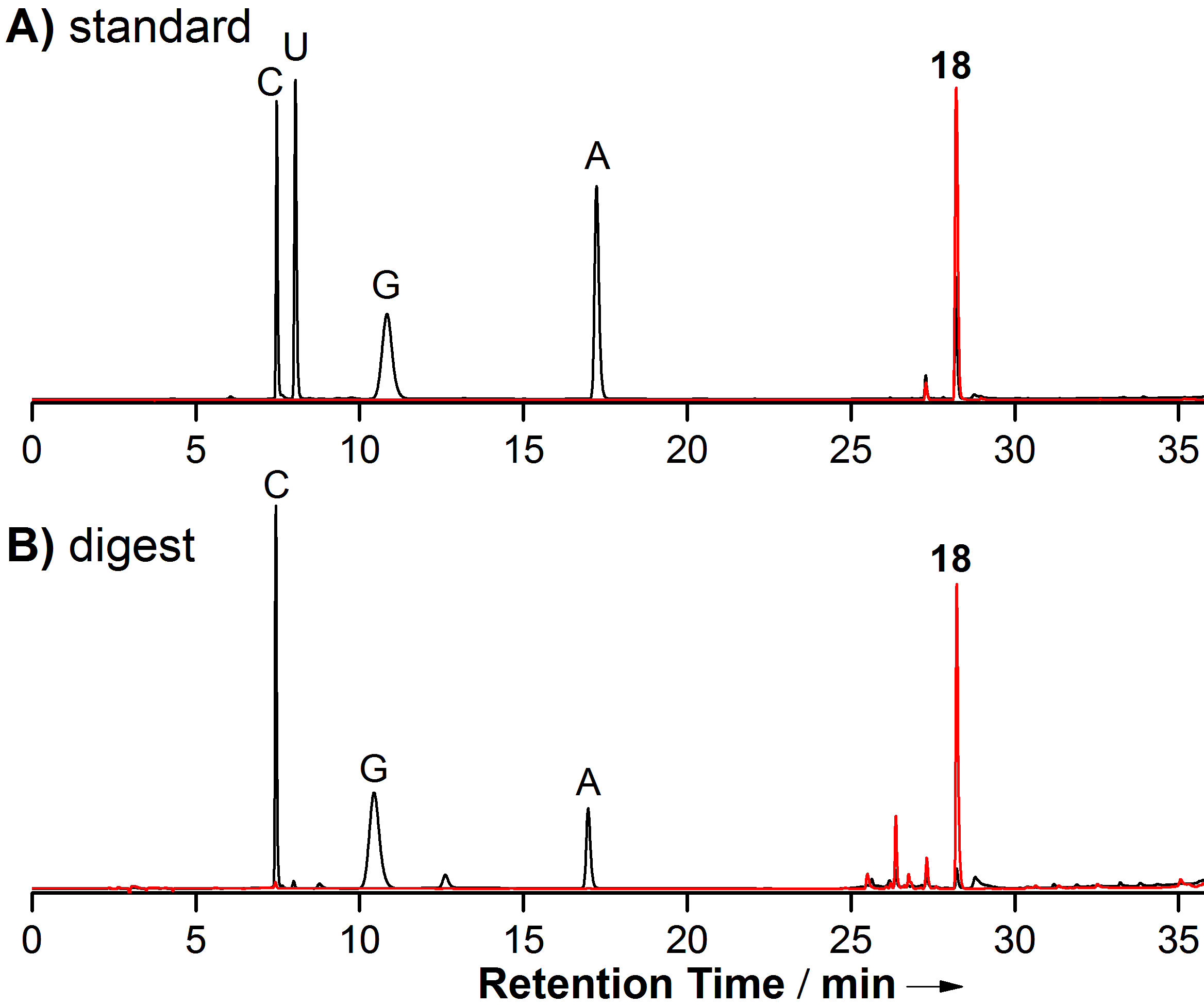


**Supplementary Figure S7.** RP-HPLC profile of ribonucleoside products obtained after enzymatic digestion of Suzuki-coupled RNA ON **17aʹ**. (**A**) A mix of natural ribonucleosides (C, U, G, A) and 2-vinyl benzofuran modified-uridine **18** at 260 nm (black line) and 338 nm (red line). *Trans* form of 5-(benzofuran-2-yl)vinyl uridine (**18**) was synthesized by Suzuki coupling reaction between IU (**1**) and boronic ester **17** (see below for the synthesis procedure). **18** absorbs at 260 nm and 338 nm. (**B**) Transcript **17aʹ** digest at 260 nm (black line) and 338 nm (red line). The retention time and strong absorbance at 338 nm confirmed the presence of *trans* form of the modified nucleoside **18** in RNA ON **17aʹ**. Mass analysis of individual fractions of the digested RNA ON confirmed the identity of the natural and modified nucleosides (Table S2).

**Supplementary Table S2.** MALDI-TOF mass analysis of HPLC fractions of RNA ON **17aʹ** digest.

| HPLC fraction | Calculated mass for | Found |
| --- | --- | --- |
| C | C_9_H_13_N_3_O_5_K [M+K]^+^: 282.3 | 282.0 |
| G | C_10_H_13_N_5_O_5_K [M+K]^+^: 322.3 | 322.1 |
| A | C_10_H_13_N_5_O_4_Na [M+Na]^+^: 290.2 | 290.1 |
| **18** | C_19_H_18_N_2_O_7_Na [M+Na]^+^: 409.4 | 409.0 |

**Synthesis of *trans* form of 5-(benzofuran-2-yl)vinyl uridine (18)**


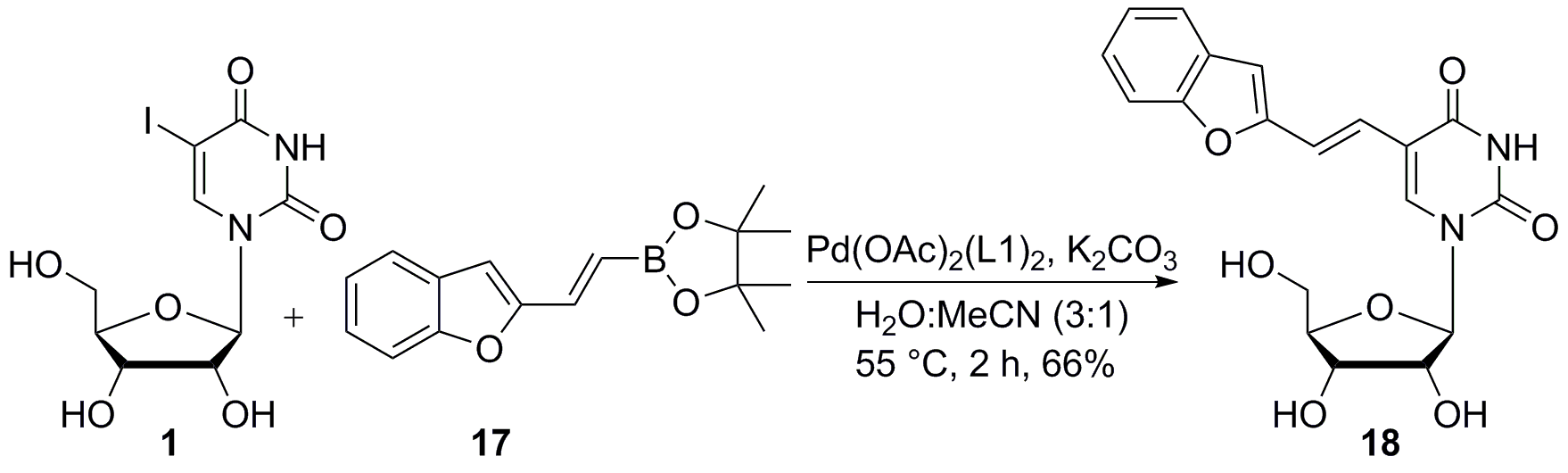


5-iodouridine **1** (229 mg, 0.62 mmol, 1 equiv.), boronic ester **17** (250 mg, 0.93 mmol, 1.5 equiv.), K_2_CO_3_ (257 mg, 1.86 mmol, 3 equiv.) were taken in a round-bottomed flask containing H_2_O (12 mL) and MeCN (4 mL). Pd(OAc)_2_(L1)_2_ (624 µL of a 50 mM stock, 5 mol%,) was added and the reaction mixture was heated at 55 °C for 2 h. After completion of the reaction, the reaction mixture was filtered through a Celite pad and was subsequently washed with MeOH. Solvent was evaporated under vacuum. The crude product was purified by silica gel column chromatography to afford the *trans* product as an off-white solid **18** (159 mg, 66%). Under these conditions *cis* isomer could not be isolated. TLC (MeOH:CH_2_Cl_2_ = 15:85); *R_f_* = 0.48; ^1^H NMR (400 MHz, *d_6_*-DMSO) δ (ppm) 11.60 (br, 1H), 8.35 (s, 1H), 7.59–7.57 (m, 1H), 7.54–7.50 (m, 2H), 7.30–7.26 (m, 1H), 7.22 (td, *J* = 7.6, 0.8 Hz , 1H), 6.99 (d, *J =* 16 Hz, 1H), 6.88 (s, 1H), 5.82 (d, *J* = 4.8 Hz, 1H), 5.46 (br, 1H), 5.32 (*_app_*t, *J* = 4.4 Hz, 1H), 5.11 (br, 1H), 4.11 (s, 1H), 4.04 (*_app_*t, *J* = 4.6 Hz, 1H), 3.90–3.87 (m, 1H), 3.78–3.74 (m, 1H), 3.65–3.61(m, 1H); ^13^C NMR (100 MHz, *d_6_*-DMSO) δ (ppm) 162.1, 155.1, 154.1, 149.6, 139.8, 128.9, 124.6, 123.1, 123.0, 121.0, 115.8, 110.7, 110.0, 104.9, 88.3, 84.7, 73.8, 69.3, 60.5; HRMS: m/z Calcd. for C_19_H_19_N_2_O_7_ [M+H]^+^ = 387.1192, found = 387.1189; *ɛ_260_* = 4200 M^-1^cm^-1^, *ε_338_* = 11960 M^-1^cm^-1^.

**
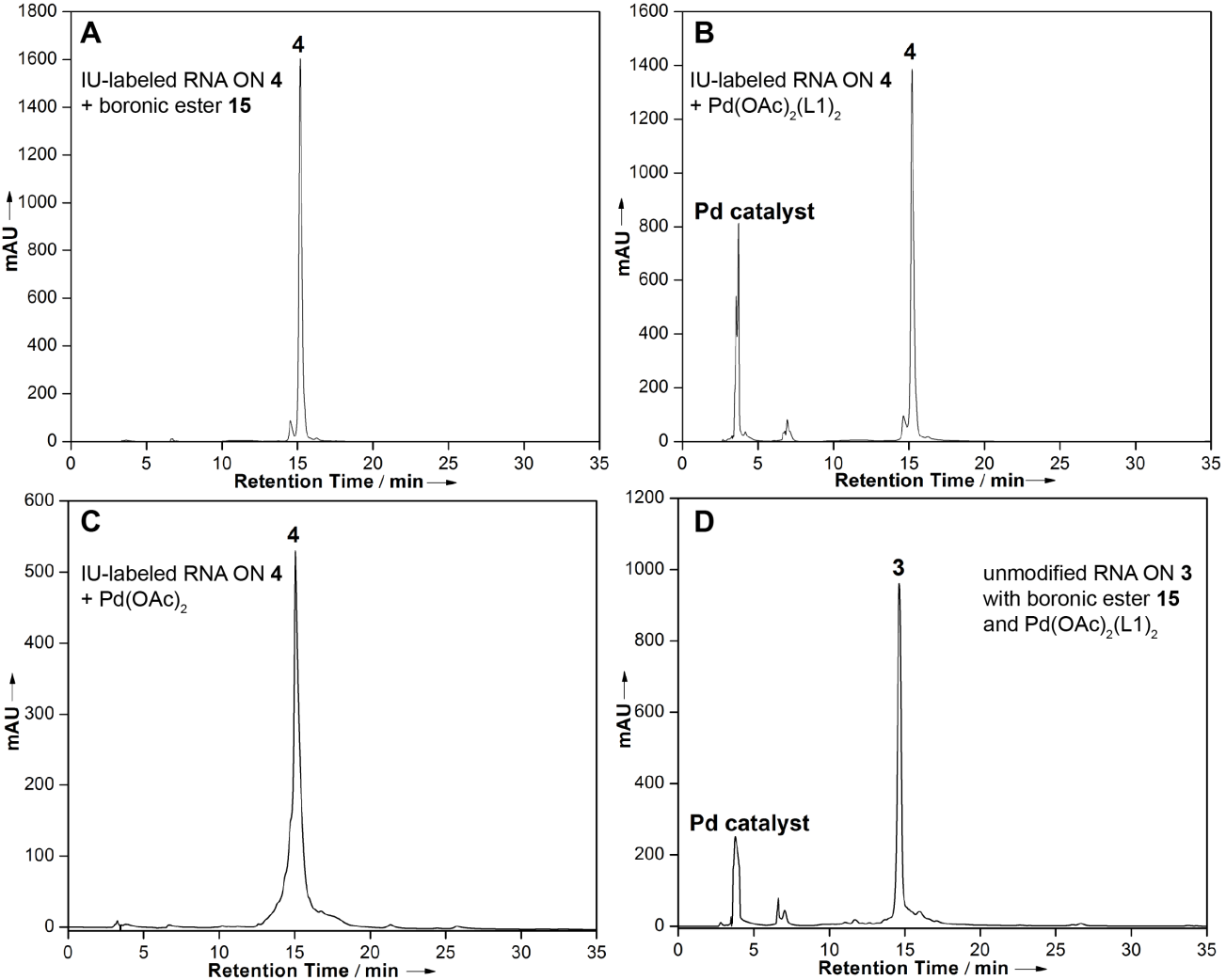
**

**Supplementary Figure S8.** Control Suzuki coupling reactions with IU-labeled RNA ON **4** (5 nmol) and unmodified RNA ON **3** (5 nmol) were performed under similar conditions. (**A**) Reaction mix containing RNA ON **4** and boronic ester **15**. (**B**) Reaction mix containing RNA ON **4** and Pd(OAc)_2_(L1)_2_. (**C**) Reaction mix containing RNA ON **4** and Pd(OAc)_2_. (**D**) Reaction of unmodified RNA ON **3** in the presence of boronic ester **15** and catalytic system Pd(OAc)_2_(L1)_2_. Formation of Suzuki coupled RNA ON product was not observed in all the cases.


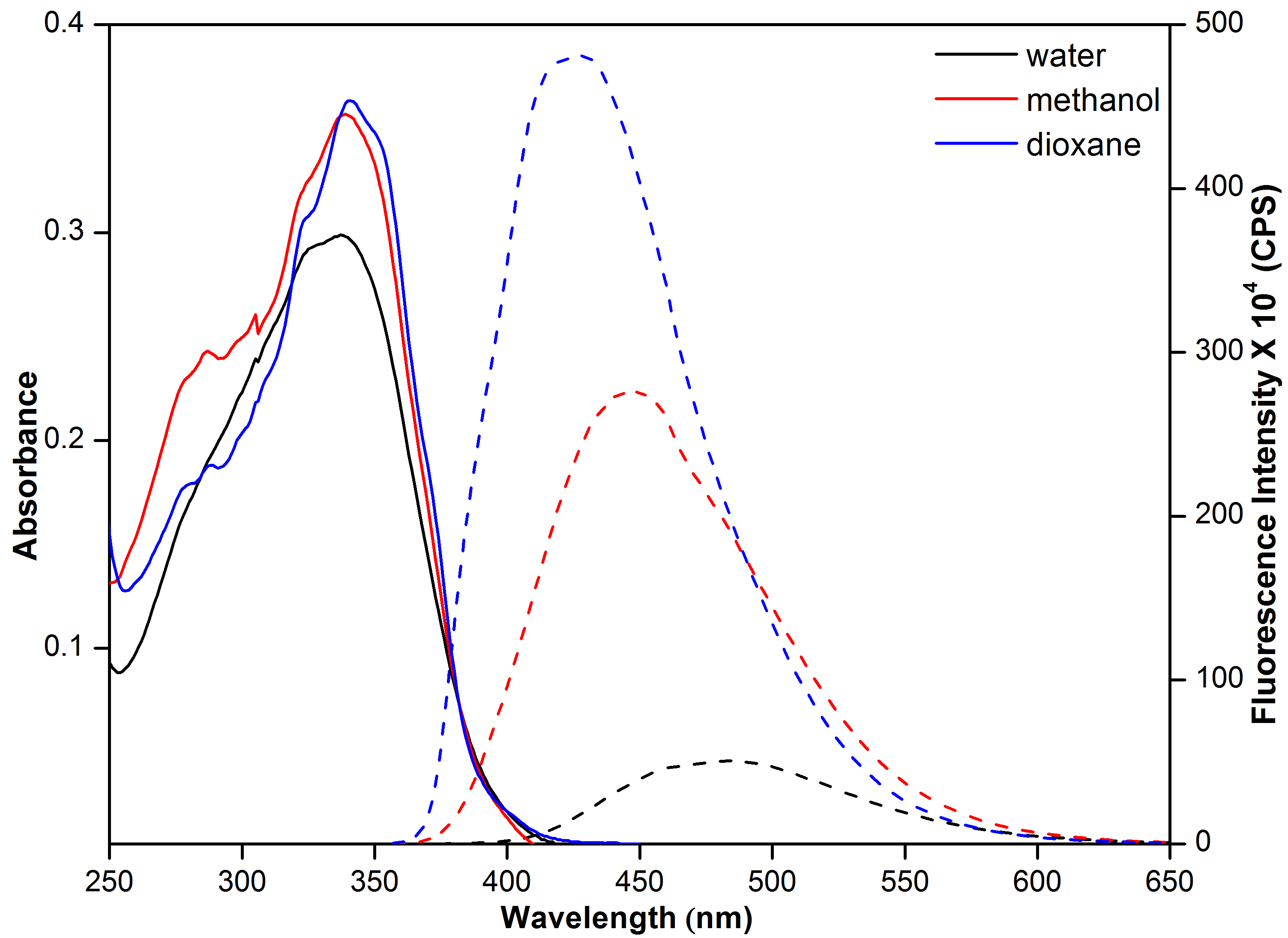


**Supplementary Figure S9.** Absorption (25 μM, solid line) and emission (5.0 μM, dashed line) spectra of 5-(benzofuran-2-yl)vinyl uridine **18** in solvents of different polarity. For absorption and emission study, the samples contained 2.5 and 0.5% DMSO, respectively. The samples were excited at longest absorption maximum (Table S3) with an excitation and emission slit width of 2 nm and 3 nm, respectively.

**Supplementary Table S3.** Photophysical properties of modified nucleoside **18** in different solvents.

| Solvent | *λ_max_*^[a]^  (nm) | *λ_em_*  (nm) | *I_rel_*^[b]^ | Stokes shift  (cm^-1^) | *Φ*^[c]^ |
| --- | --- | --- | --- | --- | --- |
| water | 338 | 483 | 1.0 | 8882 | 0.028 |
| methanol | 339 | 447 | 5.0 | 7127 | 0.097 |
| dioxane | 341 | 427 | 9.7 | 5906 | 0.149 |

^[a]^ Longest absorption maximum is given. ^[b]^ Emission intensity relative to the intensity in water is given.

^[c]^ Quantum yield of **18** in different solvents relative to 2-aminopurine as a standard was determined using the following equation.

Where s is the standard, x is **18**, A is the absorbance at excitation wavelength, F is the area under the emission curve, *n* is the refractive index of the solvent, and$\Phi F$ is the quantum yield.

Standard deviation for Φ is ≤0.004.

**5. NMR spectra**

^1^H NMR of IUTP **2** in D_2_O (400 MHz). Peaks corresponding to triethylammonium acetate buffer are present.


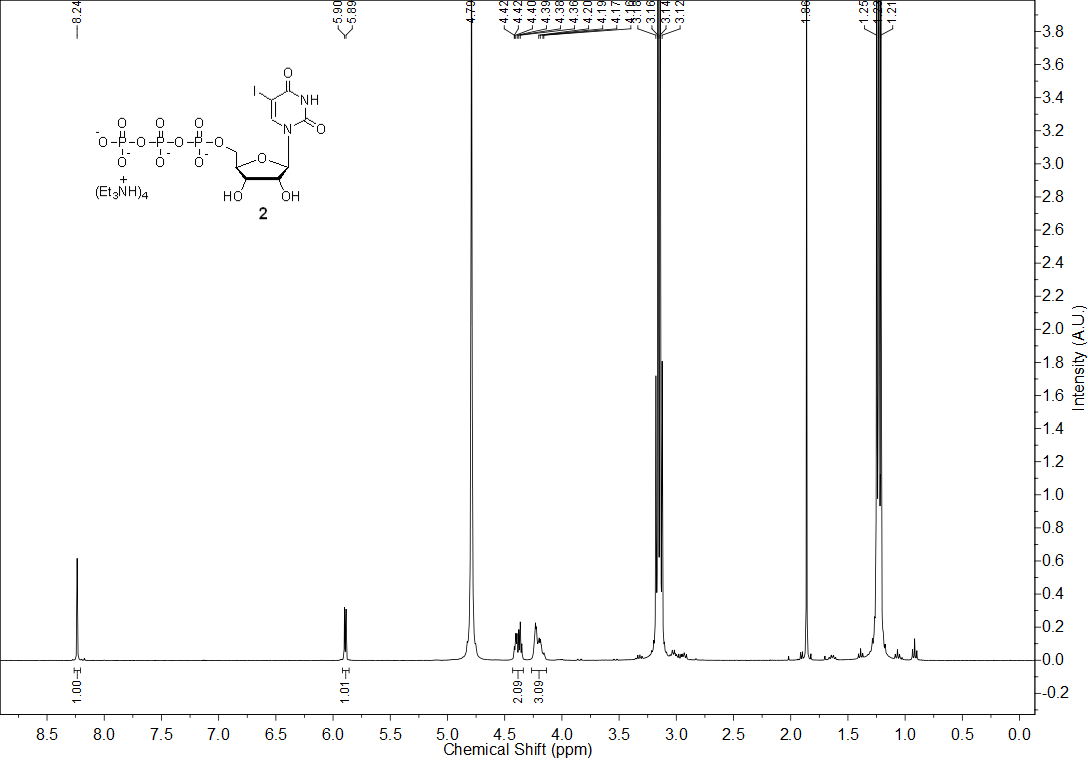


^31^P NMR of IUTP **2** in D_2_O (162 MHz)


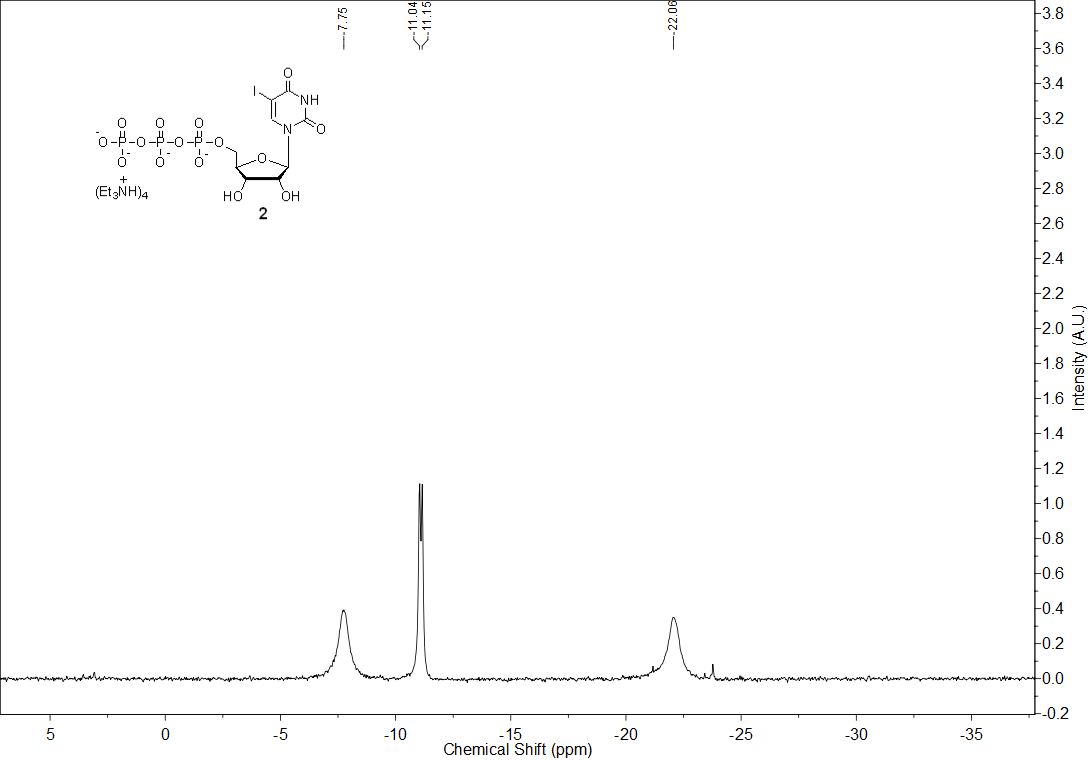


^1^H NMR of the compound **9** in CDCl_3_ containing 0.03% (v/v) TMS (400 MHz)


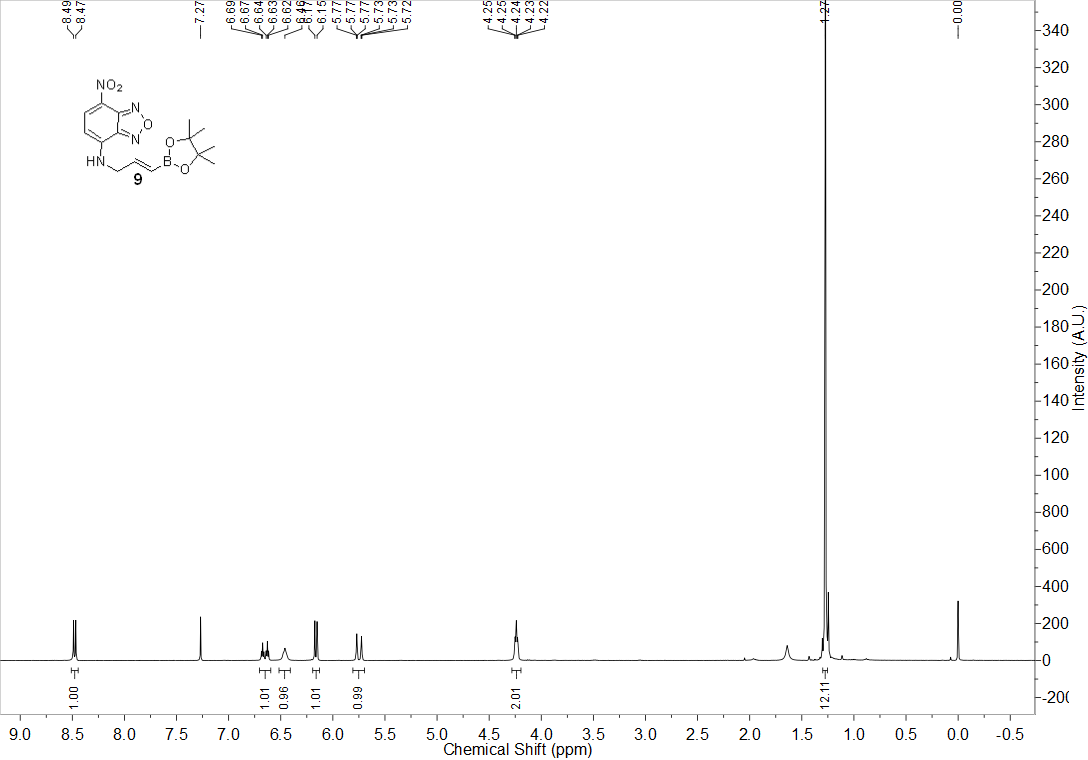


^13^C NMR of the compound **9** in CDCl_3_ containing 0.03% (v/v) TMS (100 MHz)


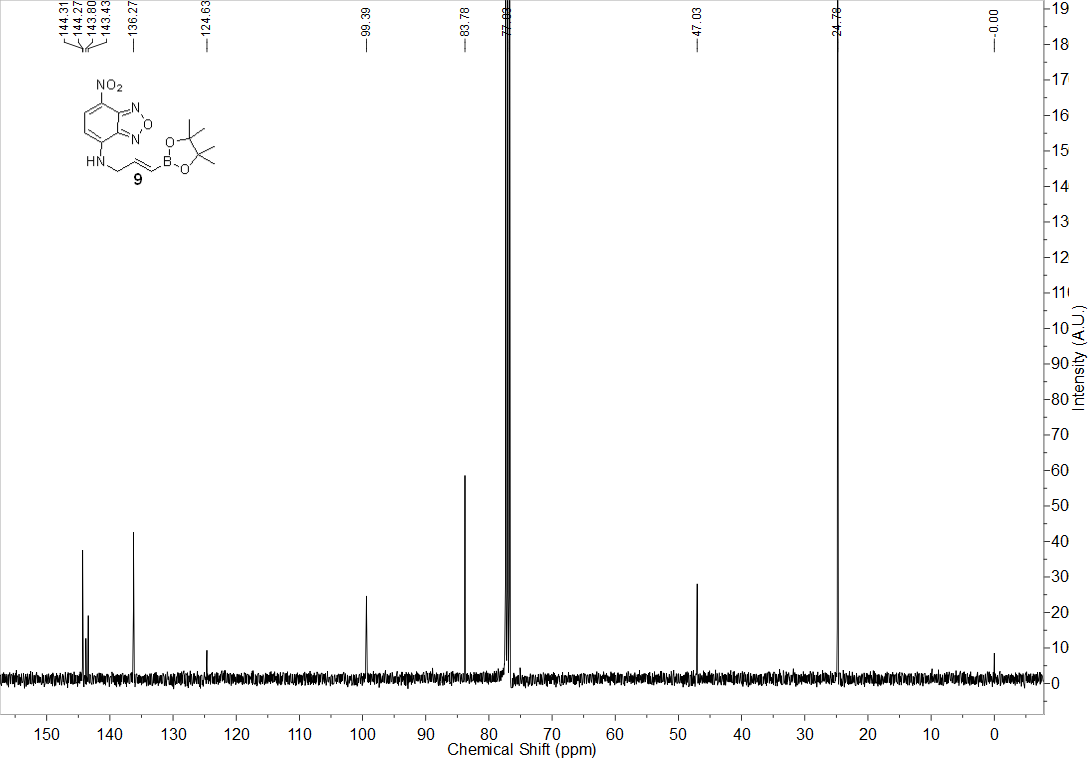


^1^H NMR of the compound **10** in CDCl_3_ containing 0.03% (v/v) TMS (400 MHz)


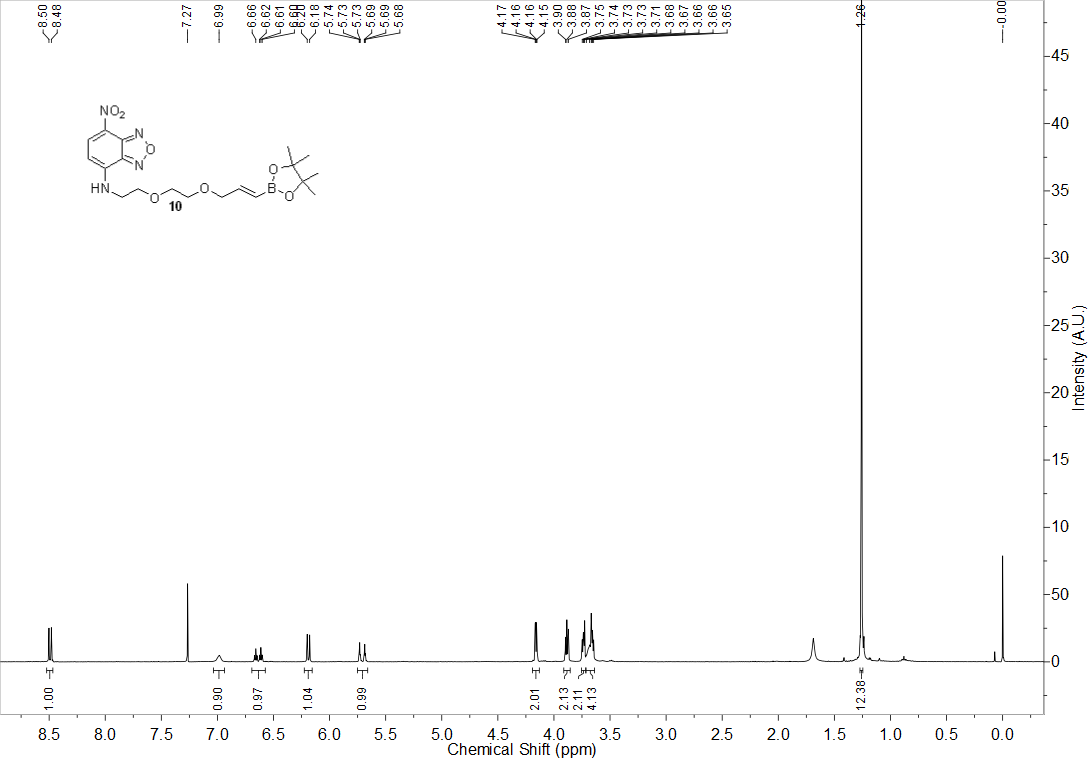


^13^C NMR of the compound **10** in CDCl_3_ containing 0.03% (v/v) TMS (100 MHz)


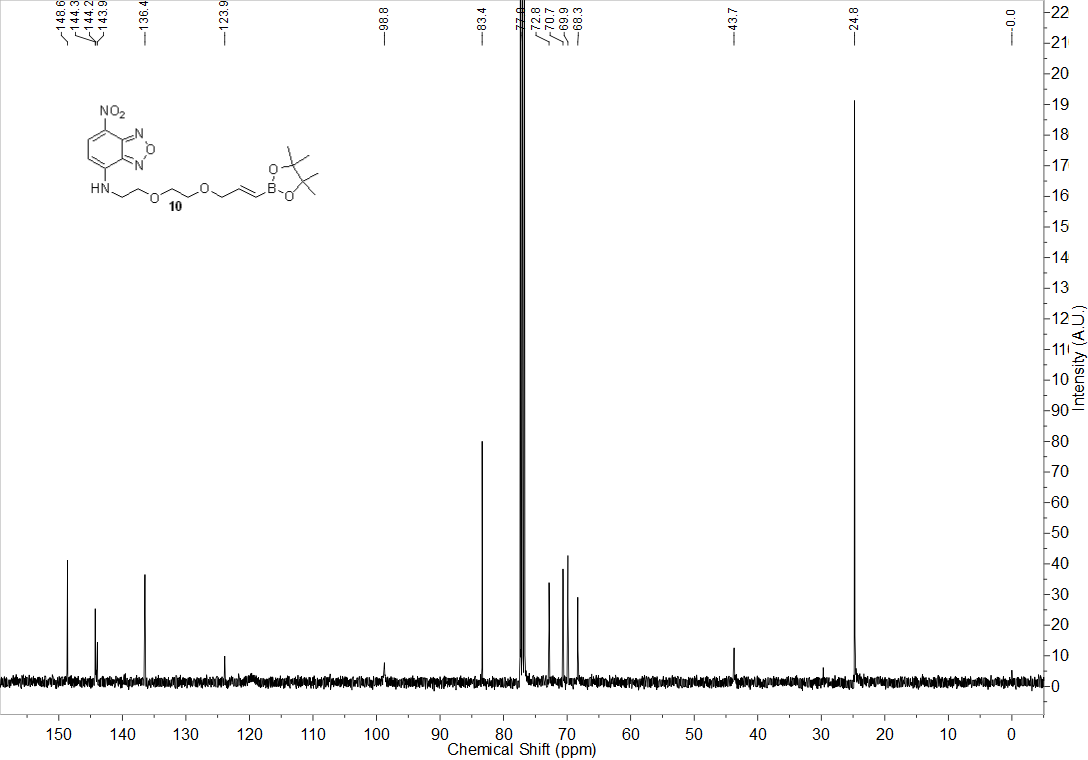


^1^H NMR of the compound **11** in CDCl_3_ containing 0.03% (v/v) TMS (400 MHz)


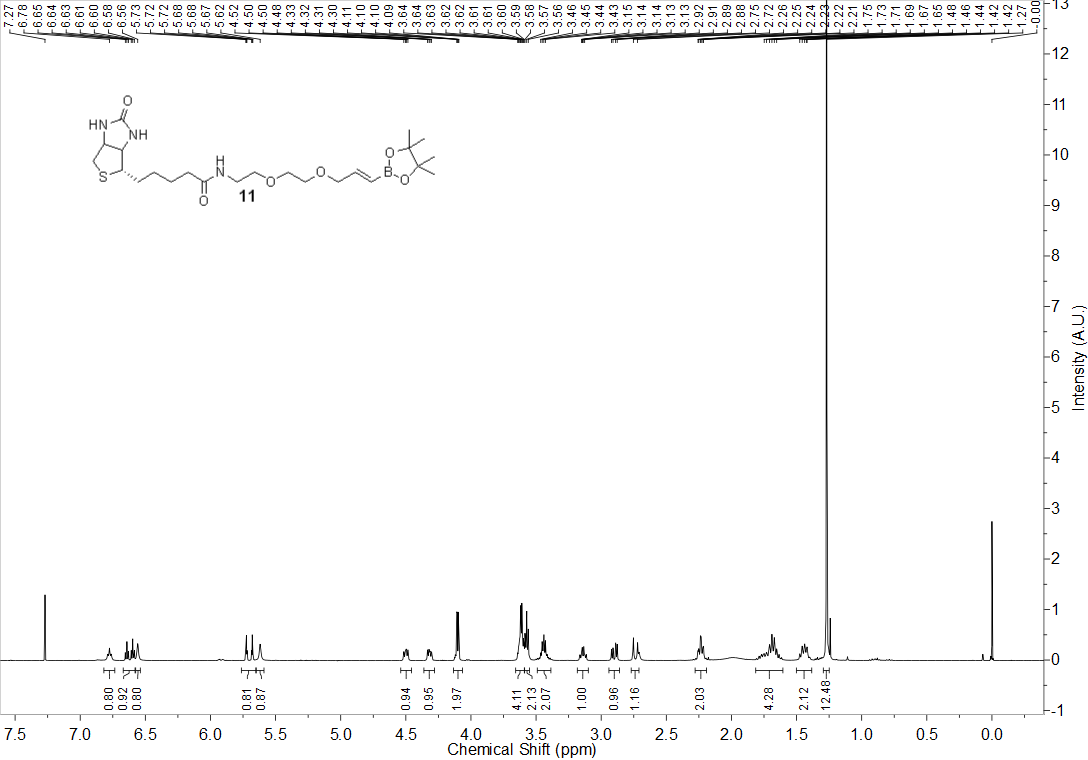


^13^C NMR of the compound **11** in CDCl_3_ containing 0.03% (v/v) TMS (100 MHz)


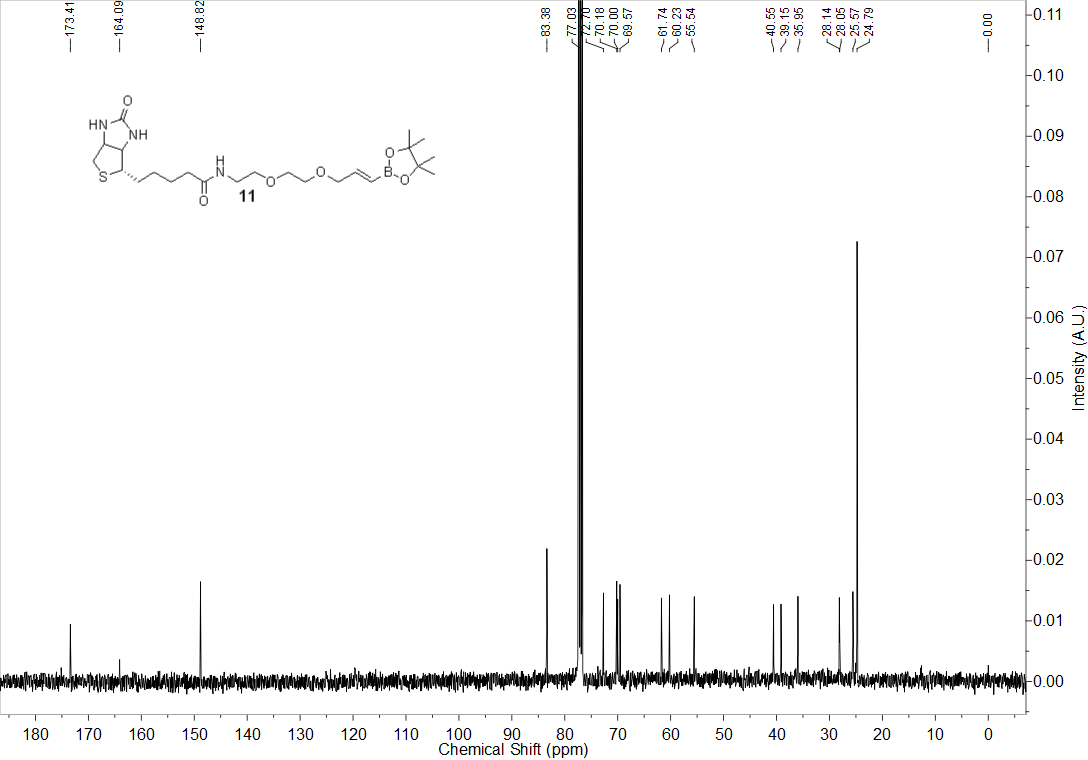


^1^H NMR of the compound **16** in CDCl_3_ (400 MHz)


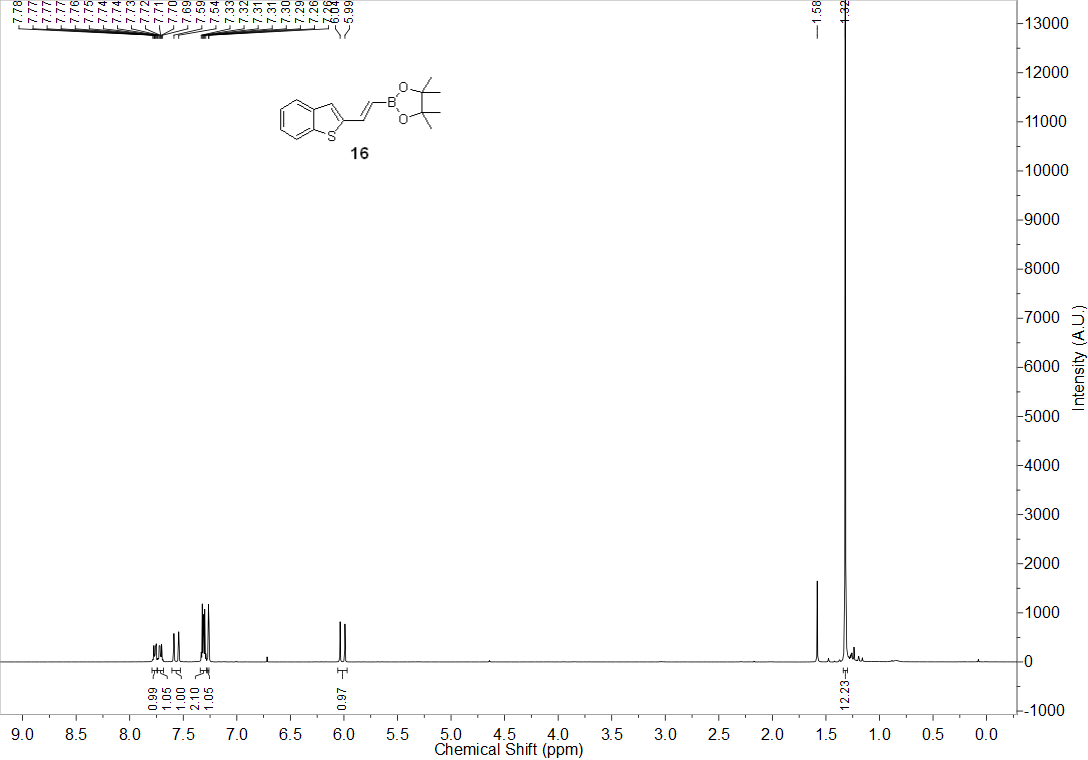


^13^C NMR of the compound **16** in CDCl_3_ (100 MHz)


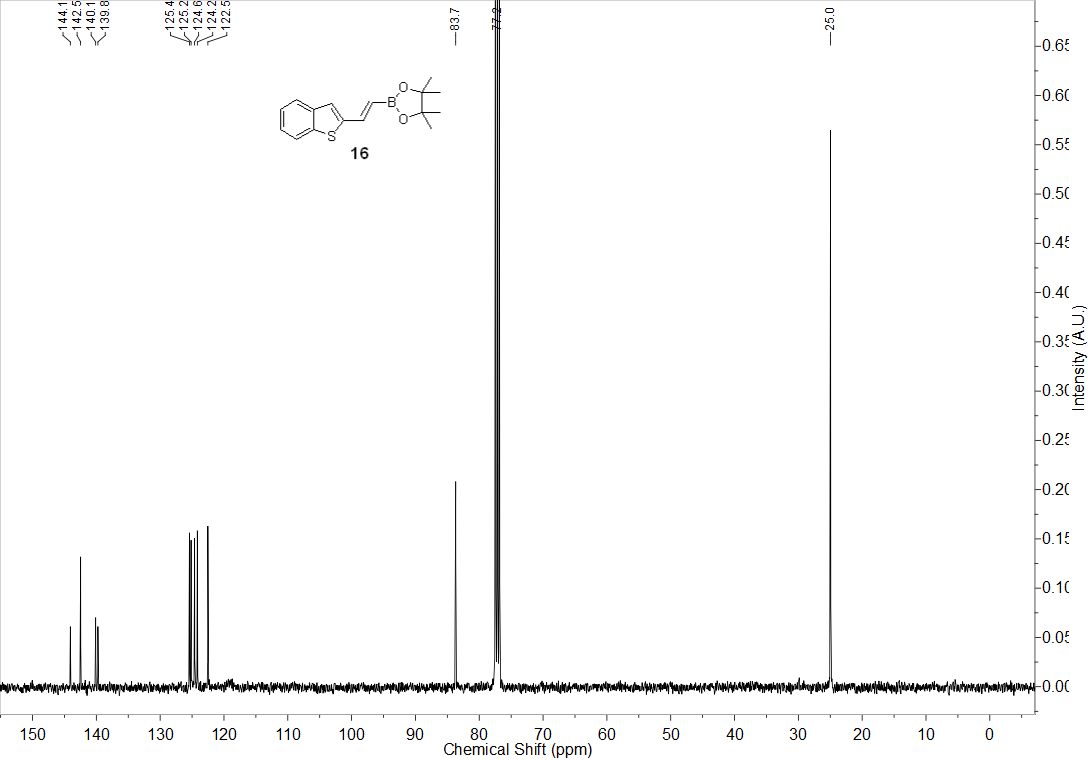


^1^H NMR of the compound **17** in CDCl_3_ (400 MHz)


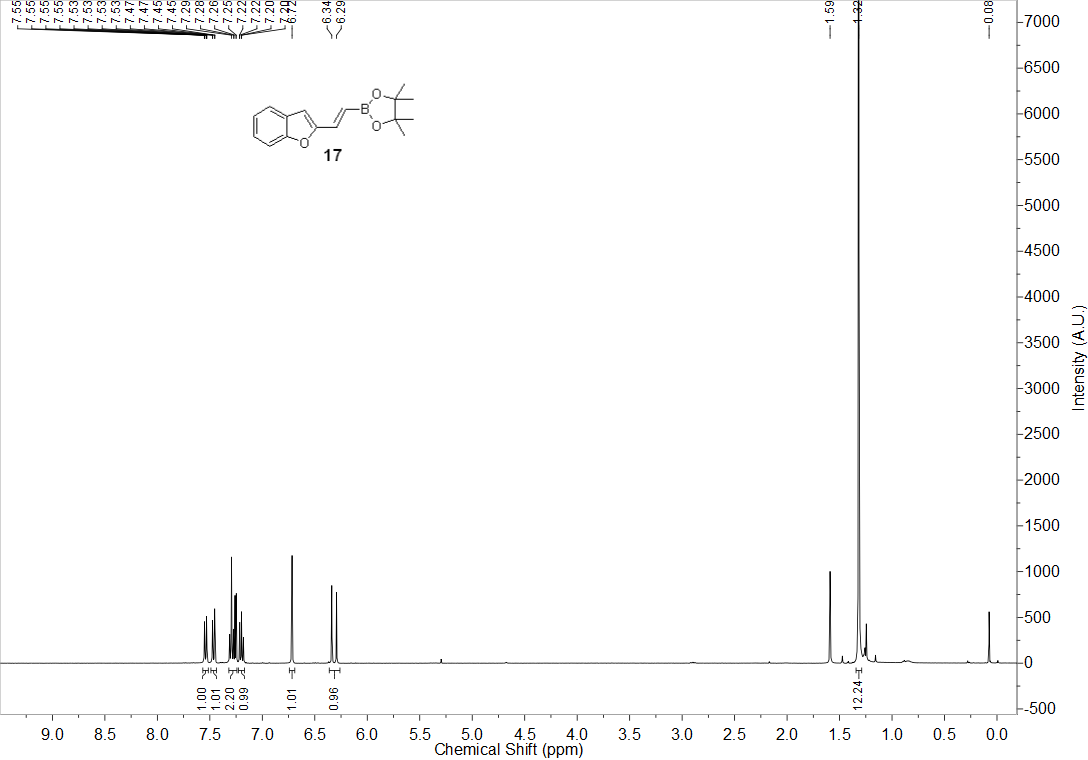


^13^C NMR of the compound **17** in CDCl_3_ (100 MHz)


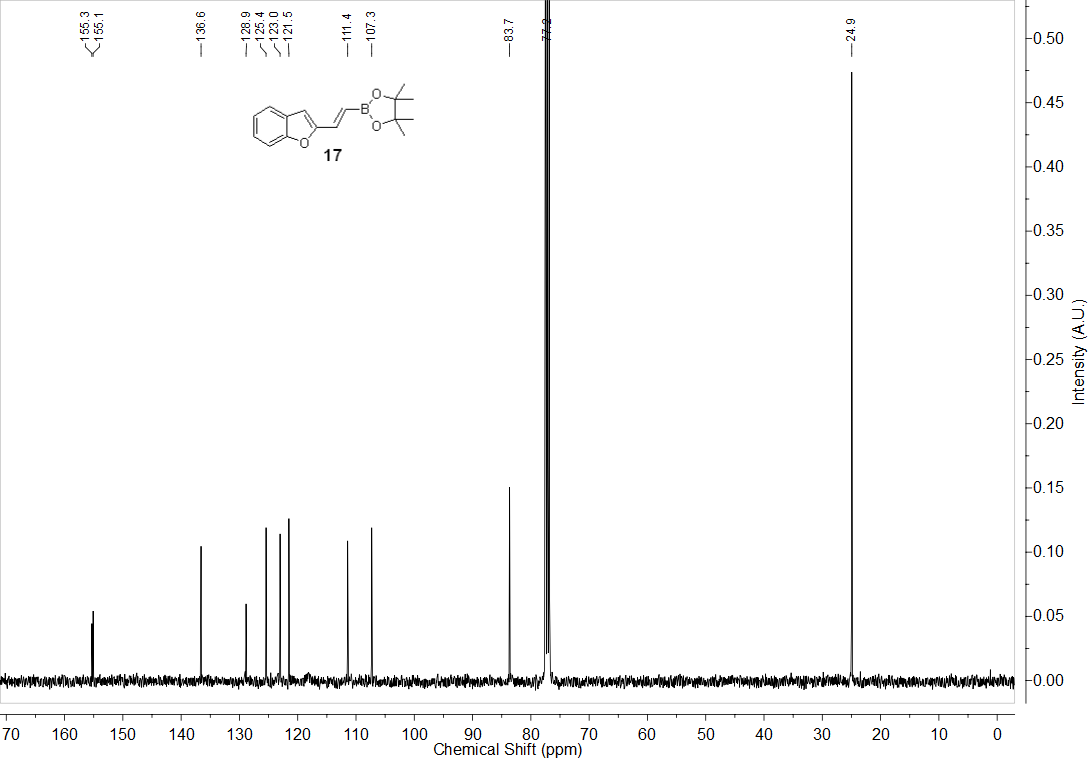


^1^H NMR of the compound **18** in *d_6_*-DMSO (400 MHz)


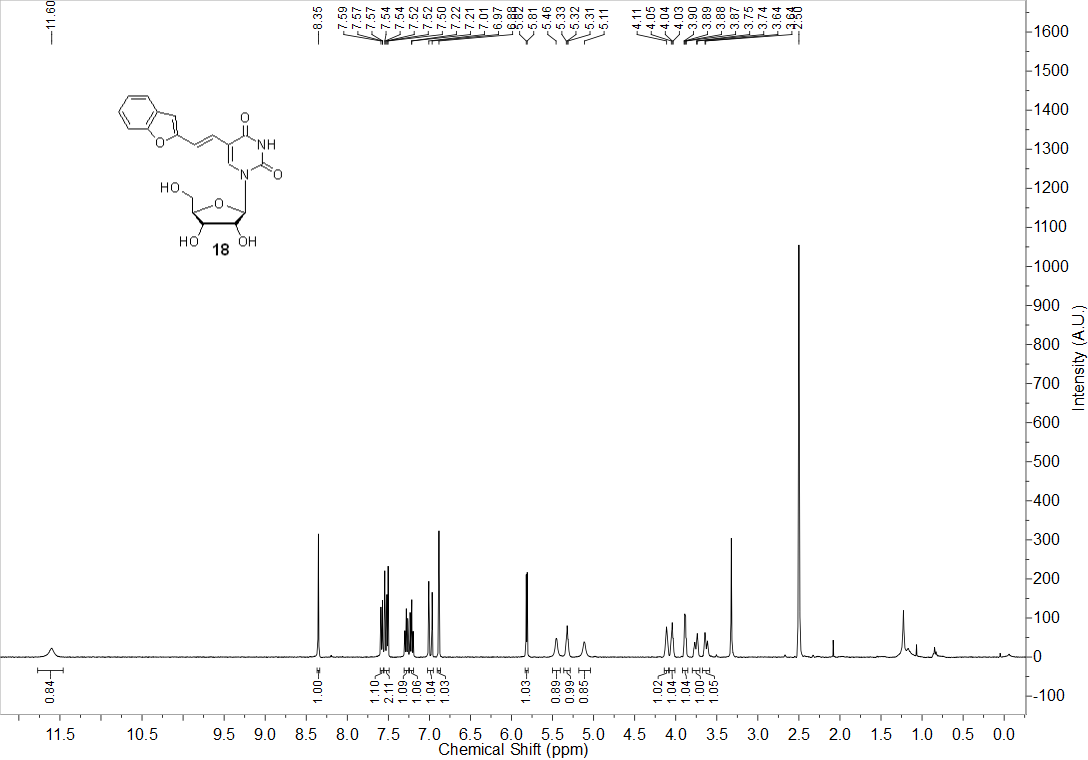


^13^C NMR of the compound **18** in *d_6_*-DMSO (100 MHz)

**
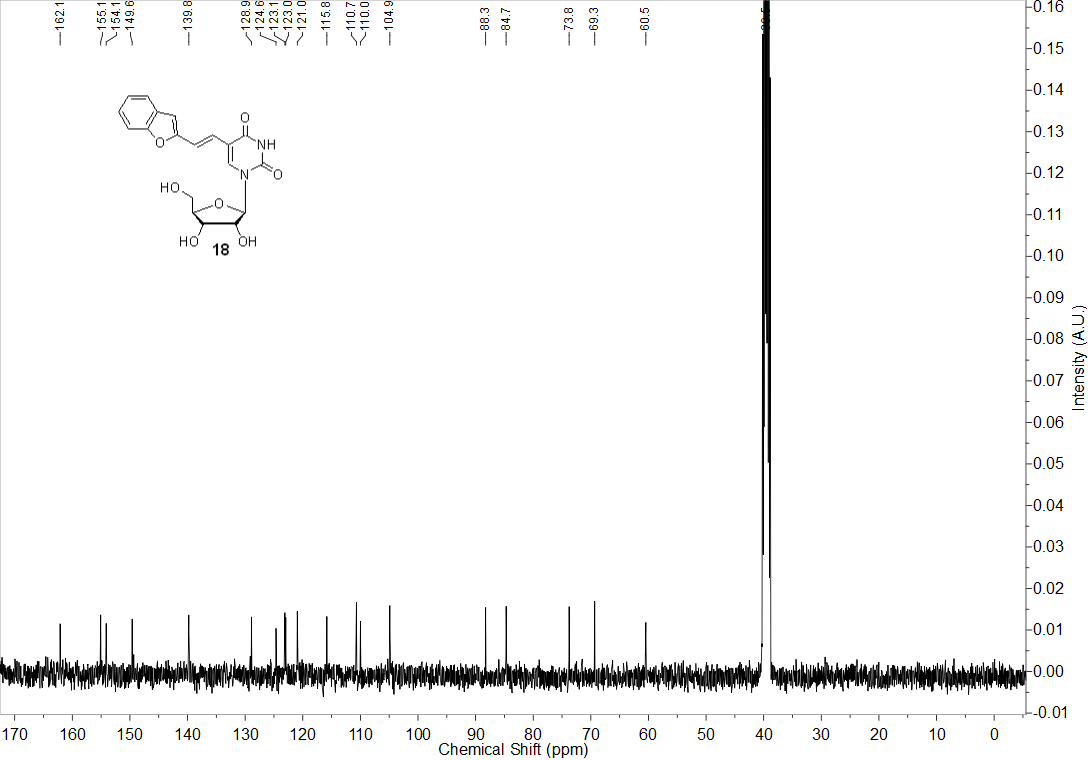
**

**6. References**

1. Flasche, W., Cismas, C., Herrmann, A. and Liebscher, J. (2004) Lipophilic nucleosides by Sonogashira coupling. *Synthesis*, **14**, 2335–2341.

2. Boon, W. R. (1952) 6- Dichloro-2-dimethylamino pyrimidine *J. Chem. Soc*., 1532.

3. Moffatt, J. G. (1964) A general synthesis of nucleoside-5′ triphosphates. *Can. J. Chem.*, **42**, 599–604.

4. Pesnot, T., Tedaldi, L. M., Jambrina, P. G., Rostac, E. and Wagner, G. K. (2013) Exploring the role of the 5-substituent for the intrinsic fluorescence of 5-aryl and 5-heteroaryl uracil nucleotides: a systematic study. *Org. Biomol. Chem.*, **11**, 6357–6371.

5. Lercher, L., McGouran, J. F., Kessler, B. M., Schofield, C. J. and Davis, B. G. (2013) DNA modification under mild conditions by Suzuki–Miyaura cross-coupling for the generation of functional probes. *Angew. Chem. Int. Ed.*, **52**, 10553–10558.

6. Kottani, R., Valiulin, R. A. and Kutateladze, A. G. (2006) Direct screening of solution phase combinatorial libraries encoded with externally sensitized photolabile tags. *Proc. Natl. Acad. Sci. U.S.A.*, **103**, 13917–13921.

7. Blanchard, D. J. M., Cservenyi, T. Z. and Manderville, R. A. (2015) Dual fluorescent deoxyguanosine mimics for FRET detection of G-quadruplex folding. *Chem. Commun.*, **51**, 16829–16831.

8. Zang, H. and Larock, R. C. (2002) Synthesis of β- and γ-carbolines by the Palladium/Copper-catalyzed coupling and cyclization of terminal acetylenes J. Org. Chem., **67**, 7048–7056.
